# Peak intensity prediction in MALDI-TOF mass spectrometry: A machine learning study to support quantitative proteomics

**DOI:** 10.1186/1471-2105-9-443

**Published:** 2008-10-20

**Authors:** Wiebke Timm, Alexandra Scherbart, Sebastian Böcker, Oliver Kohlbacher, Tim W Nattkemper

**Affiliations:** 1 Applied Neuroinformatics Group, Bielefeld University, Germany; 2 Friedrich-Schiller-University, Jena, Germany; 3 Simulation of biological systems, Center for Bioinformatics Tübingen, Eberhard Karls University, Tübingen, Germany; 4 Intl. NRW Graduate School for Bioinformatics and Genome Research, Bielefeld University, Germany

## Abstract

**Background:**

Mass spectrometry is a key technique in proteomics and can be used to analyze complex samples quickly. One key problem with the mass spectrometric analysis of peptides and proteins, however, is the fact that absolute quantification is severely hampered by the unclear relationship between the observed peak intensity and the peptide concentration in the sample. While there are numerous approaches to circumvent this problem experimentally (e.g. labeling techniques), reliable prediction of the peak intensities from peptide sequences could provide a peptide-specific correction factor. Thus, it would be a valuable tool towards label-free absolute quantification.

**Results:**

In this work we present machine learning techniques for peak intensity prediction for MALDI mass spectra. Features encoding the peptides' physico-chemical properties as well as string-based features were extracted. A feature subset was obtained from multiple forward feature selections on the extracted features. Based on these features, two advanced machine learning methods (support vector regression and local linear maps) are shown to yield good results for this problem (Pearson correlation of 0.68 in a ten-fold cross validation).

**Conclusion:**

The techniques presented here are a useful first step going beyond the binary prediction of proteotypic peptides towards a more quantitative prediction of peak intensities. These predictions in turn will turn out to be beneficial for mass spectrometry-based quantitative proteomics.

## Background

Today, mass spectrometry (MS) is an indispensable technique for the analysis of proteins and peptides in the life sciences. Various approaches have been developed to allow the comparison of protein abundances in cells between different environmental states. A growing number of studies in proteomics aim to quantitatively characterize proteomes for a better understanding of cellular mechanisms. These studies use either isotopic labeling or label-free methods for protein quantification [1].

Using isotopic labeling methods, protein mixtures are tagged with a stable isotope that can be used to tell samples apart by their mass shift and to directly compare peaks from different samples. With SILAC (Stable Isotope Labeling with Amino acids in Cell culture) [2], a metabolic labeling method, labels are introduced during cell growth and division. Chemical labeling methods such as ICAT (Isotope Coded Affinity Tags) [3] and iTRAQ (Isotope Tags for Relative and Absolute Quantification) [4] introduce the label into peptides after proteolytic digestion. These methods allow accurate quantification relative to the tagged sample at the expense of additional costly and time-consuming experimental processing steps. Enzymatic labeling with ^18^O [5] during or after proteolytic digestion is another technique that avoids the complications that chemical labeling may cause but can be applied if metabolic labeling is not possible. However, labeling efficiency differs between peptides, which causes difficulties when comparing abundances between different proteins.

In contrast, label-free methods directly use the signal intensities or spectral counts to estimate peptide abundances. But peak intensities also depend on peptide ionization efficiencies, which are influenced by a peptide's composition and the chemical environment. In other words, the sensitivity of a mass spectrometer varies between peptides. Therefore, two peptides with identical abundance will generally lead to different peak intensities. In a recent review of label-free LC-MS, America and Cordewener [6] state that "Normalisation of peptide abundance data is probably the most essential for improvement of the quantitative accuracy of the experiment." Absolute quantification with very high accuracy using label-free methods is possible through the use of reference peptides [7], an example being AQUA (Absolute Quantification of Proteins) [8]. But again, such methods require significant experimental effort.

Consequently, label-free techniques are routinely used only for differential quantification, that is, the determination of concentration ratios between samples.

Nonetheless, label-free methods have several intrinsic advantages over labeling techniques. Obviously, they do not require labor- and cost-intensive labeling. Also, there is no fundamental limit to the number of samples that can be compared. Unlike labeling techniques, label-free methods do not increase the mass spectral complexity. They have the potential to analyze a higher range of protein concentrations and to achieve a higher proteome coverage.

There exist two fundamentally different experimental setups for label-free quantification using MS: in both cases, proteins are digested and peptides are separated using Liquid Chromatography (LC). In the first case, the LC is directly coupled to an ElectroSpray Ionization (ESI) mass spectrometer, which allows a simple experimental setup and a rapid analysis of separated peptides. In the second case, LC fractions are spotted and analyzed using Matrix-Assisted Laser Desorption Ionization (MALDI) MS [9-11]. Using MALDI MS has certain advantages such as a more efficient data-dependent analysis: because the sample portions from the LC can be stored for several days and reanalyzed when necessary, it is possible to acquire fragmentation ion spectra for all MS parent ions that are of interest. Spectra are easier to interpret and compare because mostly singly charged ions are produced and detected.

If an estimate of the peptide-specific sensitivity of the mass spectrometer were possible, this would allow the use of label-free techniques for absolute quantification. For this, different machine learning techniques have been proposed. Lu *et al*. [12] estimate the detectability of peptides for the APEX method, i.e. the probability of a peptide being observed in a spectrum at all. The authors evaluate different classifiers for this purpose, and find that bagging with a forest of random decision trees produces the best results. The predicted values are then used to enhance the spectral counts-based, uncorrected abundance estimation by about 30%. Tang *et al*. [13] use two-layer neural networks to classify peptides into detectable and undetectable. They derive a Minimum Acceptable Detectability for Identified Proteins (MDIP), a cutoff value that maximizes the sum of true positives and true negatives. The MDIP is shown to increase as protein abundance decreases, which, according to the authors, could be utilized to improve quantification. For both studies, data is acquired using LC coupled to ESI. Mallick *et al*. [14] introduce the term *proteotypic *for peptides that are observed in more than 50% of the spectra they are expected in. The authors classify peptides from four different MS setups (PAGE-MALDI, PAGE-ESI, MUDPIT-ESI and MUDPIT-ICAT) into proteotypic and non-proteotypic peptides using a Gaussian mixture model, and achieve a cumulative accuracy of up to 90%.

Both Lu *et al*. [12] and Tang *et al*. [13] use predicted detection probabilities to correct peptide abundances. In both cases, the authors utilize detection probabilities as if these probabilities really were peak intensities. But for peak intensity correction, it seems much more appropriate to *predict peak intensities *directly. Gay *et al*. [15] classify peptides into observed and unobserved ones and, in addition, directly predict peak intensities via regression. Unfortunately, this study is flawed by the fact that the same peptides were used in the training and test sets, to make up for the small size of the dataset.

For our initial evaluation, we do not use MS measurements of LC-separated peptides; instead, proteins are separated via 2D polyacrylamide gel electrophoresis (2D-PAGE). Separated probes usually contain only a single protein that is subsequently digested and analyzed by MALDI Time of Flight (TOF) mass spectrometry. In this experimental setup, given a correct preparation and only one protein in the digested gel spot, all peptides in a spectrum have exactly the same abundance. Therefore, the peptide-specific sensitivity of the mass spectrometer can be accessed by comparing peak intensities in every such spectrum. A peptide-specific correction factor can then be calculated by dividing one over the corresponding peak intensity. It is understood that one cannot experimentally determine the peptide-specific sensitivity for all possible peptides.

In this study, we investigate if peak intensities of peptides in MALDI-TOF MS spectra can be predicted with machine learning (ML) techniques. We do not predict the probability of a certain peptide to be detectable but instead predict its normalized peak intensity directly. This is an important step to facilitate the enhancement of label-free quantification accuracy. We reach a prediction accuracy of *r *= 0.66 for across-dataset prediction, where *r *is the Pearson correlation between predicted and observed intensities. Our datasets stem from 2D-PAGE separated proteins, but clearly, our results can directly be applied to enhance the quantification accuracy of MALDI-based LC-MS experiments [9-11]. A study by Mallick *et al*. [14] shows that a slightly simpler problem, the prediction of proteotypic peptides, can be done successfully for LC-MS experiments. We are therefore hopeful that regression approaches like the one proposed here may be successful for LC-MS data, too.

Let *s *be the sequence of a peptide we have identified, and let *I *be the intensity of the MS peak corresponding to this peptide. Instead of directly using *I *as an estimate for the relative peptide concentration, we apply ML to compute a predicted intensity *pI*(*s*) of the peptide solely from the peptide sequence. We can now calculate a corrected peptide intensity *I' *= 1/*pI*(*s*)·*I *that replaces *I *in subsequent steps of the analysis. Here, 1/*pI*(*s*) is the peptide-specific correction factor. If the peak intensity estimate *pI*(*s*) is accurate then *I' *is a better estimate than *I *of the relative peptide concentration.

## Methods

We use two sets of MALDI-TOF mass spectra to generate the datasets for this study. These were measured on a Bruker Ultraflex instrument (Bruker Daltonics, Bremen, Germany) using proteins extracted from *Corynebacterium glutamicum *[16]. The proteins were separated by 2D gel electrophoresis, then digested into peptide fragments with trypsin prior to MS analysis. The corresponding peptide sequences were derived from protein identification using MASCOT peptide mass fingerprinting [17] and an in-house database containing *C. glutamicum *protein sequences. For the smaller dataset *A*, both the selection of spectra and determination of search parameters for the MASCOT identification were done manually, while for dataset *B*, both were done automatedly.

For dataset *A*, spectra were manually selected from a previously unpublished set of spectra. Search parameters were chosen manually by an expert and included fixed (carbamidomethylation of cysteine) and variable modifications (oxidation of methionine), and no missed cleavages were allowed. The list of spectra was filtered for the 20% of spectra with the best MASCOT score and the largest difference from the second best hit, resulting in 62 spectra being used for further analysis. Of 27 identified proteins, 15 were present in multiple spectra. For dataset *B*, spectra were run through fully automated MASCOT peptide mass fingerprinting search with 42 different sets of search parameters. These included with and without oxidation of methionine, tolerance within {50, 100, 150, 200, 250, 500, 750} ppm, and up to {0, 1, 2} missed cleavages allowed. The resulting list was filtered automatically to fulfill the following properties: a) protein mass in range [8000, 12000] Da, b) pI between 4 and 7 because of the 2D gel used, c) highest MASCOT protein hit score above 65, and d) sequence coverage above 15% using the search parameters that produced the highest score. Spectra with more than one high-scoring hit were also removed. Application of this protocol left 182 spectra for further analysis. Of 125 identified proteins, 35 were present in more than one spectrum. To summarize the differences, *A *can be considered a small, carefully chosen dataset while *B *is larger and of lesser overall quality. An overview table can be found in additional file 1: datasets and a histogram showing the number of spectra per protein in additional file 2: spectranumbers. The additional files 3 and 4: dataset_a_protein_list and dataset_b_protein_list show lists of the identified proteins. We give a short outline of our spectra preprocessing pipeline; details are deferred to additional file 5: preprocessing). After de-noising with a Savitzky-Golay filter [18], baseline correction, and removal of noise peaks, peak intensities are extracted from the spectra. We unfold isotopic distributions by adding up all peak intensities of isotope peaks that belong to the same peptide. The resulting list of peaks is matched against masses calculated from a theoretical tryptic digestion. The matching of sequences to peaks is straightforward in this case because the peptide sequences are known. We ignore missed cleavages and variable modifications in the matching process. We allow for a mass error of up to 1 Da to make up for calibration errors. In case of multiple peaks inside the allowed mass error range, the one closest to the theoretical mass is chosen.

In dataset *A*, for 371 out of 535 expected peptides, we detected the corresponding peak in the mass spectrum. In dataset *B*, 1023 out of 1994 predicted peaks were detected. For most peptide sequences, only one peak intensity measurement exists: in dataset *A*, 49.2% of peptide sequences are unique, whereas in dataset *B*, this is true for 70.2% of the peptides.

Abundances differ between spectra. To use intensities from different spectra together in one dataset, we need to normalize them. Since the amount of protein in each spectrum is unknown, we introduce two normalizations for peak intensities. We compare the performance of both normalizations in our experimental evaluation.:

• *Normalization by corrected mean ion current (mic)*. The intensity of a peak *p *is scaled by the mean ion current, i.e. the mean of all baseline-corrected measurements *C*_1_,...,*C*_*N *_in the spectrum:

$$I_{p}^{mic}=\frac{I_{p}}{\frac{1}{N}\sum_{i=1}^{N}C_{i}},$$

where *I*_*p *_denotes the raw intensity of peak *p *after peak extraction. Here, index *i *runs over all raw values (i.e. not only peptide peaks) of the spectrum the peptide was found in. By doing so, we take into account intensities of unmatched peptides as well as differences in the overall sensitivity of the detector.

• *Normalization by sum of peptide peak intensities (sum)*. The intensity of a peak *p *is scaled by the sum of all matched peptide's peak intensities *i *= 1,...,*P *to yield

$$I_{p}^{sum}=\frac{1000\cdot I_{p}}{\sum_{i=1}^{P}I_{i}},$$

where *I*_*i *_denotes the intensity of the *i*^*th *^peptide peak after peak extraction. A similar approach has been used by Radulovic *et al*. [19]. The factor 1000 is used for numerical reasons.

Some peptides are present in more than one spectrum and, hence, these peptides show more than one peak intensity value. Most learning architectures do not cope well with different target values per input.

Therefore, we calculate an *α*-trimmed mean with *α *= 50% for peptides with more than three target values, and a mean for peptides with two or three target values. As we will see below, this also allows us to estimate the potential prediction accuracy. As a final preprocessing step, we logarithmize intensities with a natural logarithm such that the resulting error becomes additive, which stabilizes the variance [1,20,21], and the values themselves become approximately normal distributed (see Q-Q plots in additional file 6: qqplots).

We can now state the peak intensity prediction problem as a supervised learning problem. A training set Γ = {(**x**, *y*)_*j*_, *j *= 1,..., *N*} consists of input-output pairs (**x**, *y*) where **x **∈ ℝ^*t *^is an input peptide feature vector, and *y *∈ ℝ is the normalized intensity we want to predict (target value). Obviously, this is a (nonlinear) regression problem and a wide range of techniques can be applied.

When calculating target intensities for each peptide, we assume that all proteins were correctly identified, we assume perfect digestion, and we ignore variable modifications for all steps following identification. We are aware that this is not perfectly true in reality: There are a few missed cleavages, variable modifications were found during the database search, and we can not totally exclude the possibility of misidentification, even though MASCOT thresholds were chosen to practically exclude this case. In this sense, our datasets can be seen as "imperfect" and cleaner datasets could be used. But if intensity prediction is possible using this imperfect, partly erroneous and noisy data, results will only improve when cleaner datasets are available. To show that we in fact learn to predict intensities from peptide sequences, we shuffled the assignment of peptide sequences to target values and found that no prediction is possible for the shuffled dataset; see below.

The same arguments hold for the effect of *ion suppression *[22]: the signal of a compound is suppressed in the presence of other compounds that compete for ionization. Intensity prediction could take this effect into account if each peptide occurred in multiple spectra with all possible combinations of other peptides, and if there was no contamination. However, such a dataset is impossible to acquire. Knowing this, we neglect the fact that peptide peak intensities depend not only on the peptide's constitution, but also on the combination of other peptides present. We consider this effect an additional noise component our method has to cope with. In any application of our method, this effect would always be unknown, and we aim at a realistic estimation of the performance of our method.

### Machine learning methods

We selected two complementary non-linear regression architectures. *ν*-Support Vector Regression (*ν*-SVR) [23] has excellent generalization abilities and copes well with high-dimensional input data [24]. However, it is difficult to interpret its models, and parameter-tuning can be time-consuming. The second learning architecture, the Local Linear Map (LLM) [25] is less accurate if applied to higher-dimensional feature spaces but is very efficient (i.e. fast training and adaptation to addition data, and low memory-usage). It is more transparent and can be used for peptide prototyping as shown in [24]. Both architectures represent different principles of learning non-linear regression functions. There exist cases where a linear model outperforms more sophisticated regression models. Therefore, we apply a simple linear model (LM) [26] for comparison. Other methods have been tested and perform similarly to or worse than those presented here (data not shown).

#### *ν*-Support Vector Regression (*ν*-SVR)

Support vector machines are a class of learning algorithms that are designed to implicitly transfer input feature vectors into a high-dimensional feature space where classes are linearly separable and the optimal linear decision boundary can be calculated. In practice, however, it depends on the choice of a kernel function and the data whether linear separability is actually achieved. For support vector *regression*, the *ε*-insensitive loss function |*y *- *f*(**x**)|_*ε *_= max {0, |*y *- *f*(**x**)| - *ε*} was introduced by Vapnik *et al*. [27]. Here, errors are only considered if they are higher than *ε *for some fixed *ε *> 0. Since the choice of an appropriate *ε *can be difficult, the *ν*-SVR introduced by Schölkopf *et al*. [23] finds the best *ε *automatically by minimizing a cost function. Instead, *ν*, an upper bound of the number of allowed errors and a lower bound to the number of support vectors, has to be chosen *a priori*. The *ν*-SVR generalizes an estimator for the mean of a random variable discarding the largest and smallest examples (a fraction of at most $\frac{\nu }{2}$ of either category), and estimates the mean by taking the average of the two extremal values of the remaining examples. This results in good robustness of the *ν*-SVR. Other parameters that have to be chosen are the regularization parameter *C *and kernel width *γ*. Parameter *C *controls the trade-off between the weight of errors and the complexity of the regression function. Parameter *γ *controls the width of the radial basis function $K(x,x^{\prime })=e^{-\gamma ||x-x^{\prime }|{|}^{2}}$, which we use as kernel function. We use the libsvm implementation of the e1071 package available for R [28,29].

#### Local Linear Map (LLM)

Local Linear Maps [25], a type of artificial neural net, combines an unsupervised vector quantization algorithm based on Self-Organizing Maps (SOM) [30] with supervised linear learning principles for prediction of peak intensities. The LLM can learn global non-linear regression functions by fitting a set of *local *linear functions to the training data. It has been successfully used for peptide prototyping [31] and provides a basis for peptide feature profiling and visualization.

Motivated by the SOM, an LLM consists of a set of *n*_*l *_regular ordered nodes **v**_*i*_, *i *= 1,...,*n*_*l*_, which are connected to each other via a two-dimensional grid structure, defining a neighborhood between the nodes and a topology in feature space. Each node consists of a triple $v_{i}=(w_{i}^{\text{in}},w_{i}^{\text{out}},A_{i})$. The vectors $w_{i}^{\text{in}}\in {\mathbb{R}}^{d_{\text{in}}}$ are used to build prototype vectors adapting to the statistical properties of the input data $x_{\xi }\in {\mathbb{R}}^{d_{\text{in}}}$. The vectors $w_{i}^{\text{out}}\in {\mathbb{R}}^{d_{\text{out}}}$ approximate the distribution of the target values $y_{\xi }\in {\mathbb{R}}^{d_{\text{out}}}$. The matrices $A_{i}\in {\mathbb{R}}^{d_{\text{in}}\times d_{\text{out}}}$ are locally trained linear maps from the input to the output space.

In the unsupervised training phase, the prototype vectors $w_{i}^{\text{in}}$ are adapted following the SOM learning rule: the vectors $w_{i}^{\text{in}}$ are pulled towards the input pattern **x**_*ξ *_according to the distance between the input pattern and the corresponding closest prototype in input space $w_{\kappa }^{\text{in}}$ with $\kappa =\underset{i}{\arg\min}\{||x_{\xi }-w_{i}^{\text{in}}||\}$. The learning procedure changes the weights according to a Gaussian neighborhood function *h*_*σ *_with width *σ *decreasing over grid distance *r*_*k*_(**x**, *n*_*l*_):

$$h_{\sigma }(r_{k}(x,n_{l}))=\exp\left(-\frac{r_{k}(x,n_{l})}{2\sigma^{2}}\right).$$

The learning step widths *ϵ*, *ϵ*^*A*^, *ϵ*^*out *^∈ [0; 1] for updating neighbors and *s *are decreased during training. After adapting the prototypes, a classification can be applied by assigning every input vector **x **to its closest prototype.

After unsupervised adaptation and tessellation of the input space, an input feature vector is mapped to an output by the corresponding local expert:

$$C(x)=w_{\kappa }^{\text{out}}+A_{\kappa }\left(x_{\xi }-w_{\kappa }^{\text{in}}\right).$$

The weights $w_{i}^{\text{out}}$ and the linear map **A**_*i *_are changed iteratively by the gradient descent learning rules:

$$\begin{array}{c} \Delta w_{i}^{\text{out}}=\epsilon^{\text{out}}\cdot h_{\sigma }\cdot (y_{\xi }-C(x_{\xi })),\\ \Delta A_{i}=\epsilon^{A}\cdot h_{\sigma }\cdot (y_{\xi }-C(x_{\xi }))\cdot \frac{{\left(x_{\xi }-w_{i}^{\text{in}}\right)}^{T}}{||x_{\xi }-w_{i}^{\text{in}}|{|}^{2}}. \end{array}$$

The concept of approximating nonlinear functions by fitting simple models to localized subsets of the data is related to other regression approaches like Locally-Weighted Regression (LOESS) [32] and to radial basis functions (RBF) [33]. Hastie *et al*. demonstrated the usefulness of locally linear function fitting as well [34]. We use here our own implementation of LLM as an R package.

### Linear model (LM)

A linear regression model assumes the data to have the structure **y **= **X**^*T*^**b**, which corresponds to data lying on a straight line. Here, **X **is the input data in matrix formulation, **y **the vector of target values, and **b **a vector of coefficients that have to be found when adapting the model to the data. The ordinary least squares (OLS) algorithm is applied to find the coefficients. OLS minimizes the squared differences (errors) between the model's output and the target values of the training examples.

### Feature extraction

We cannot directly use peptide sequences as input for the learning architectures, and derive numerical *feature vectors ***x**_*j *_to represent molecular features of peptide *j*. In bioinformatics, peptides are usually represented as strings over the alphabet of amino acid characters. However, a biochemist is more interested in the chemical properties of a peptide to characterize it. These paradigms motivate different feature sets:

• A 20-dimensional feature set with only **single amino acid counts (mono)**.

• A purely sequence-based 9220-dimensional **sequence feature set (seq)**. Each peptide is mapped to the 9220-dimensional vector by counting how often a certain *k*-mer appears in the sequence. Each feature vector contains 20 counts for all single amino acids (like *mono*), 400 counts for all di-peptides, 8000 counts for all tri-peptides, and two times 400 counts for terminal di-peptides at the beginning and end of the peptide sequence. Additional file 7 (substringfrequency) shows the frequency distribution of di- and tri-peptide strings in the used datasets. Here, "di-peptides" or "tri-peptides" refer to substrings of the peptide sequences, not to single molecules consisting of two or three amino acids. We will show below that the pure sequence-based feature set is often not sufficient for a decent prediction of peak intensities, what motivates the use of the next feature set.

• A 531-dimensional **chemical feature set (aa) **computed from amino acid attributes. Attributes are taken from the *amino acid index *database [35]. Each amino acid index *AA *= (*AA*_1_,...,*AA*_20_) consists of twenty real values for the twenty amino acids. Let *m*(*s*) be the number of occurrences of the amino acid *s *in the amino acid index. For a peptide *S *= *s*_1_...*s*_*n*_, the value for the corresponding feature *f *is calculated as the sum of attribute values, $f=\sum_{k=1}^{n}AA_{m(s_{k})}$. This value reflects the overall property of the peptide. There are 516 attributes in the amino acid index database, therefore we can calculate 516 such features. In addition, we use features for peptide length, mass, and numbers and fractions of acidic, basic, polar, aliphatic, and arginine residues. Finally, three features for gas-phase basicity are added to the feature vector: a) The estimated gas-phase basicity is calculated as proposed by Zhang *et al*. [36] as well as b) a sum over the residual values of the amino acids that were used for this estimation, and c) that sum scaled with the length of the peptide.

In the course of our analyses, we also evaluate what features are particularly important for the task of predicting peak intensities. This leads us to the following feature set:

• A reduced feature set resulting from forward stepwise selection on *aa *and *seq*. This **selected subset feature set (sss) **is 18-dimensional; its features can be found in Table 1. The following section explains how they were chosen.

**Table 1:** Features constituting the sss feature set

**feature ID**	**explanation**	**selected**
GB500	Estimated gas-phase basicity at 500 K (Zhang *et al*., 2004)	20
VASM830103	Relative population of conformational state E (Vasquez *et al*., 1983)	11
NADH010106	Hydropathy scale (36% accessibility) (Naderi-Manesh *et al*., 2001)	9
FAUJ880111	Positive charge (Fauchere *et al*., 1988)	6
WILM950102	Hydrophobicity coefficient in RP-HPLC, C8 with 0.1%TFA/MeCN/H_2_O (Wilce *et al*. 1995)	6
OOBM850104	Optimized average non-bonded energy per atom (Oobatake *et al*., 1985)	2
**mass**	Molecular mass of the peptide	-
**KHAG800101**	The Kerr-constant increments (Khanarian-Moore, 1980)	-
**NADH010107**	Hydropathy scale (50% accessibility) (Naderi-Manesh *et al*., 2001)	-
**ROBB760107**	Information measure for extended without H-bond (Robson-Suzuki, 1976)	-
**FINA770101**	Helix-coil equilibrium constant (Finkelstein-Ptitsyn, 1977)	-
**ARGP820102**	Signal sequence helical potential (Argos *et al*., 1982)	-

R	No. of arginine residues	20
F	No. of phenylalanine residues	20
M	No. of methionine residues	17
Q	No. of glutamine residues	5
Y	No. of tyrosine residues	4
**H**	No. of histidine residues	-

The "selected" column shows the number of times out of twenty runs of a forward stepwise selection that selected the corresponding feature. Hand-picked features are printed in bold face. Feature selection on the *aa *(above the separating line) and *seq *(below) feature set were done independently of each other. The *seq *feature set fully includes *mono*. No di- or tri-peptide string was selected consistently.

All features are centered and normalized by variance prior to training. Datasets and feature vectors are available from . Raw data and identifications are available from .

### Feature selection

Often, accuracy, speed, and interpretability can be increased by reducing the number of features. To select a few features out of hundreds, we apply a simple greedy selection method as follows: a forward stepwise selection as described in [37] (section 3.4) was applied twenty times to the *aa *and *seq *feature set of dataset A (*mic*). This method starts with the intercept and calculates a value $F_{i}=\frac{e-e_{+}}{e_{+}/(N-k-2)}$ for each feature *i*, where *e *is the prediction error of a 10-fold cross-validation with the *ν*-SVR on the current model and *e*_+ _the prediction error of the model with the additional feature *i*. *N *denotes the number of training examples, and *k *the number of features in the smaller model. The feature that gives the highest value *F*_*i *_is added to the model before the next iteration. The procedure is repeated until no feature produces an *F*_*i *_that is higher than the 95th percentile of the *F*_1,*N*-*k*-2 _distribution. For the *ν*-SVR, we used the parameters chosen by the grid search on the full feature set, since it is infeasible to repeat a grid search for each selection step. The resulting feature set depends on random partitioning during 10-fold cross-validation. Thus, each application of the selection algorithm can produce a different feature set. We selected those features that were selected most often (≥ 5 out of 20), reviewed the chosen features, and added others that might also be important for peptide-specific sensitivity of a mass spectrometer.

### Evaluation techniques

**Figure 1:**
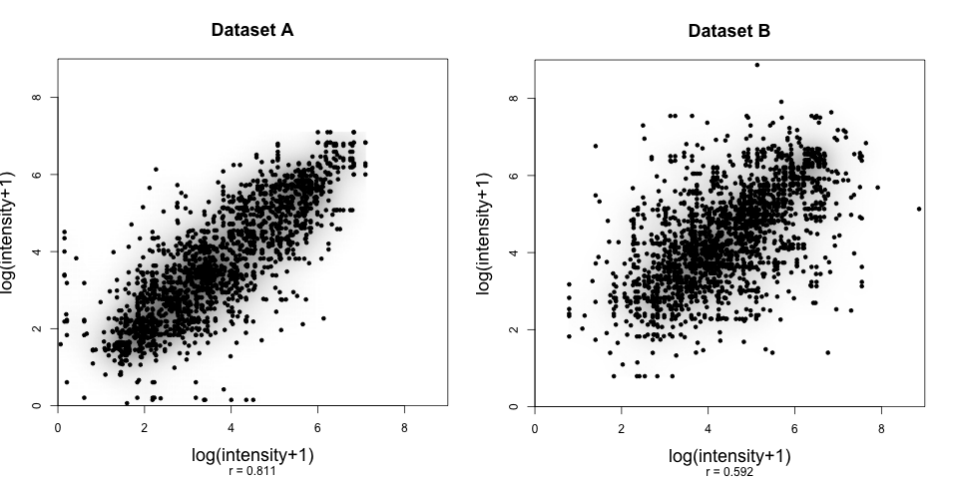
**Within-peptide variances of target values**. Scatter plots and correlation coefficients depicting the within-peptide peak intensity variance between runs for all peptides of both datasets (*left*: dataset *A*, *right*: dataset *B*). The recorded correlations can be considered as upper bounds of the achievable prediction performance if single measurements are used. The corresponding plots with trimmed mean values can be found in the additional file 7: tmbetweenpeptidecorrelation.

To evaluate correlation coefficients recorded in the following, we want to estimate how good our prediction accuracy can possibly get. To do so, we analyze the variation of intensity values for each peptide. Recall that many peptides are present in more than one mass spectrum, and one peptide sequence may correspond to multiple peak intensity values. If we compute the correlation of normalized intensity values for all peptides with multiple values, we find a correlation coefficient of *r *= 0.81 for dataset *A*, and *r *= 0.59 for dataset *B *(Fig. 1). To generate training data for our learning approaches, we compute target values as the trimmed mean of intensities for peptides with more than three observations, which reduces the effect of outliers. Comparing the target values of each peptide sequence to its trimmed mean for all peptides with multiple target values, we record a correlation coefficient of *r *= 0.92 for dataset *A *and *r *= 0.82 for *B*. The corresponding scatter plots are shown in additional file 8: tmbetweenpeptidecorrelation. Since we use trimmed mean intensities as input, these correlation values can be interpreted as "upper bounds" for correlation coefficients any machine learning technique may achieve using this data. We are confident that for other datasets, even better prediction accuracies are possible.

We determine the best parameter set for each regression model using 10-fold cross-validation. We make sure that the ten sets contain disjunct peptide sequences. A grid search over the parameter space is performed to determine optimal parameters. The 10-fold cross-validation is applied for each parameter set. The best parameter set is the one with highest mean Pearson correlation coefficient (*r*) between target and predicted value over all ten test sets. The mean squared error (MSE) has also been calculated. However, it is not comparable between datasets without additional normalization, whereas the Pearson correlation coefficient is independent of the scale used. In addition, in this case, the parameter set with the lowest MSE is always the same as the one with the highest *r*, or very close to it.

For *ν*-SVR, the grid search runs over *C *∈ {*e*^-3^, *e*^-1^,...,*e*^13^} and *γ *∈ {*e*^-13^, *e*^-11^,...,*e*^5^} in steps of *e*^2^, and *ν *∈ {0.2, 0.3,...,0.8} in steps of 0.1. The parameters sampled by the LLM are prototypes *n *∈ {1, 2, 3, 6, 10, 15, 25}, width of the neighborhood function *σ *∈ {5, 2, 1, 0.4}, linear vs. exponential decrease of learning step size, and number of learning iterations *t *∈ {10, 50}.

The final model is built by retraining the whole dataset with the optimal parameter set determined in the model selection step. Since the parameters may be adapted to the data used for cross-validation, we estimate the performance on new data by validating the prediction accuracy on the other dataset: The model trained on *A *is validated on *B*, and vice versa. Both datasets have 193 peptides in common, therefore we also validate across datasets with these 193 peptides excluded. The MSE of this across-dataset validation can be reduced by normalizing by variance and centering the data. Pearson's correlation coefficient is not affected by such operations.

## Results and discussion

In order to assess the methods described above, we performed a series of experiments testing various aspects. We compare the prediction performance of three types of learning architectures on two different MS datasets for four different feature sets. Performance is evaluated via 10-fold cross-validation, and we assess the generalization performance by predicting each dataset with a model trained on the other dataset. Prediction results for *mic *normalization are shown in Table 2. We first present results on the best performing predictors and then analyze the influence of the individual components in more detail.

**Table 2:** Overview of Pearson's correlation coefficients using *mic *normalization

**validation**	**dataset**	**feature set**	***ν*-SVR**	**LLM**	**LM**
10-fold CV	A	*aa*	0.66	0.52	0.60
		*sss*	0.66	0.67	0.51
		*mono*	**0.68**	0.64	0.52
		*seq*	0.57	0.34	0.34
	
	B	*aa*	0.53	0.46	0.49
		*sss*	**0.55**	0.53	0.49
		*mono*	0.47	0.53	0.48
		*seq*	0.44	0.27	0.41

across datasets	A	*aa*	0.65	0.52	0.14
		*sss*	0.63	0.59	0.47
		*mono*	**0.66**	0.57	0.45
		*seq*	0.46	0.21	0.40
	
	B	*aa*	0.45	0.24	0.01
		*sss*	**0.50**	0.44	0.39
		*mono*	0.45	0.39	0.32
		*seq*	0.32	0.05	0.28

	A	*aa*	0.58	0.47	0.00
		*sss*	0.58	0.55	0.41
		*mono*	**0.61**	0.52	0.39
across datasets		*seq*	0.37	0.21	0.22
	
without duplicates	B	*aa*	0.44	0.42	0.00
		*sss*	**0.53**	0.46	0.40
		*mono*	0.46	0.44	0.32
		*seq*	0.32	0.00	0.03

Values "0.00" indicate that the correlation coefficient was in the range (-0.005,0.005). The best value in each section is printed in bold face.

### Best performance

The normalization by corrected mean ion current (*mic*) generally has a slight advantage over the normalization by sum of peptide peak intensities (*sum*), while other observations were identical for both normalization types. Therefore, results and scatter plots for *sum *normalization are deferred to additional files. See Table 3 for a summary of all additional files with scatter plots.

**Table 3:** Summary table for additional scatter plots

	**DS**	**normalization**	***ν*-SVR**	**LLM**
10-fold CV	A	*sum*	additional file 10: asumsvrcross	additional file 11: allmcross
		*mic*	additional file 12: amicsvrcross	additional file 11: allmcross
	
	B	*sum*	additional file 13: bsumsvrcross	additional file 14: bllmcross
		*mic*	additional file 15: bmicsvrcross	additional file 14: bllmcross

across datasets	A	*sum*	additional file 16: asumsvrvalidation	additional file 17: allmvalidation
		*mic*	additional file 18: amicsvrvalidation	additional file 17: allmvalidation
	
	B	*sum*	additional file 19: bsumsvrvalidation	additional file 20: bllmvalidation
		*mic*	additional file 21: bmicsvrvalidation	additional file 20: bllmvalidation

This table summarizes the scatter plots available as additional files, and in which file a certain plot can be found. The LLM scatterplots for the *seq *feature set as well as all LM scatter plots are not been included because the results (correlation values) are poor. "DS" abbreviates "dataset".

**Figure 2:**
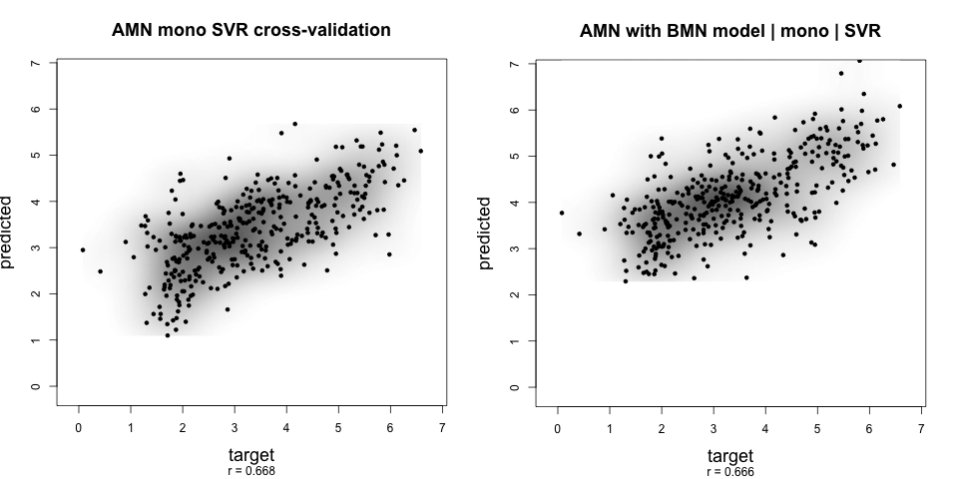
**Scatter plot target vs. predicted values**. Prediction results for dataset *A *with the *ν*-SVR indicate that peak intensity prediction is feasible. *Left*: Cross-validation on dataset *A*. *Right*: Prediction using a model parameter-tuned on dataset *B*. *r *denotes the Pearson's correlation between target and predicted values. Plots for dataset *B *and the other feature sets are shown in additional files. A summary of all additional files showing scatter plots is presented in Table 4.

Among all combinations, the **best prediction result **is achieved using the *ν*-SVR algorithm on dataset *A *with *mic *normalization and the *mono *feature set (single amino acid counts), shown in Fig. 2. Here, 10-fold cross-validation yields an overall correlation of *r *= 0.68. In the across-dataset validation, the correlation coefficient is only slightly reduced to *r *= 0.66, or *r *= 0.61 when peptides present in both datasets were excluded. *ν*-SVR, using chemical (*aa*) or selected subset (*sss*) feature sets results in prediction accuracy almost as good as for the *mono *feature set; see Table 2, as well as additional files of *A *and *B *scatter plots referred to in Table 3. These correlations are significant and show that we can predict peak intensities with statistical learning methods.

**Figure 3:**
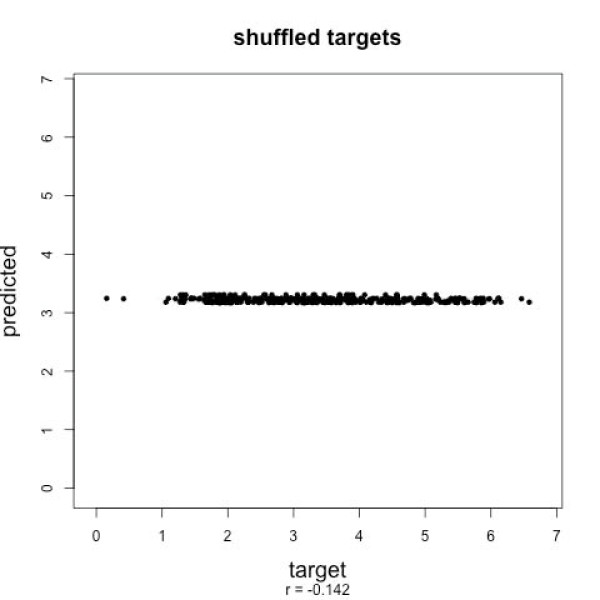
**Prediction results with randomly shuffled sequences**. When assigning randomly shuffled sequences to the target values of dataset *A*, prediction by *ν*-SVR shows no correlation in 10-fold cross-validation. This indicates that we are picking up the true signal, i.e. the predicted values are correlated to the peptide sequence.

To show that predicted values are an actual signal related to peptide sequences, and not some random pattern the learning machines find in the data, we randomly **shuffle **the assignment of peptide sequences to peak intensities. No good correlation can be observed in cross-validation when evaluating shuffled datasets: From dataset A we generated 100 datasets with randomly permuted target values. For each of the 100 shuffled datasets, we trained a *ν*-SVR with sss features and parameters optimized using another shuffled dataset. The best correlation with this dataset was *r *= 0.20. For the 100 datasets, we reach a mean correlation coefficient of *r *= *-*0.14. None of the shuffled datasets generated for a good correlation coefficient (standard deviation below 0.044). See Fig. 3 for an exemplary scatter plot. This is a clear indication that we are picking up the true signal, that is, the predicted intensities are correlated to the peptide sequence.

The scatter plots of target vs. predicted values in Fig. 2 are typical for dataset *A*. The cross-validation plot shows a point cloud that is almost diagonal and shows considerable spread especially for low values. The across-dataset prediction plot (right side of Fig. 2) shows that values of *A *are systematically predicted too high when the model trained and parameter-tuned on dataset *B *is used. An analysis of the statistical properties of the target values of both datasets reveals that *B *has a higher mean and its distribution is more skewed towards higher values. This difference can be explained by the fact that both datasets were wet-lab processed by different persons, dataset *B *by multiple persons even, and they were analyzed with different settings of the mass spectrometer. In applications of our method, an additional normalization step should be applied that accounts for these differences.

### Dependence on peak intensity

**Figure 4:**
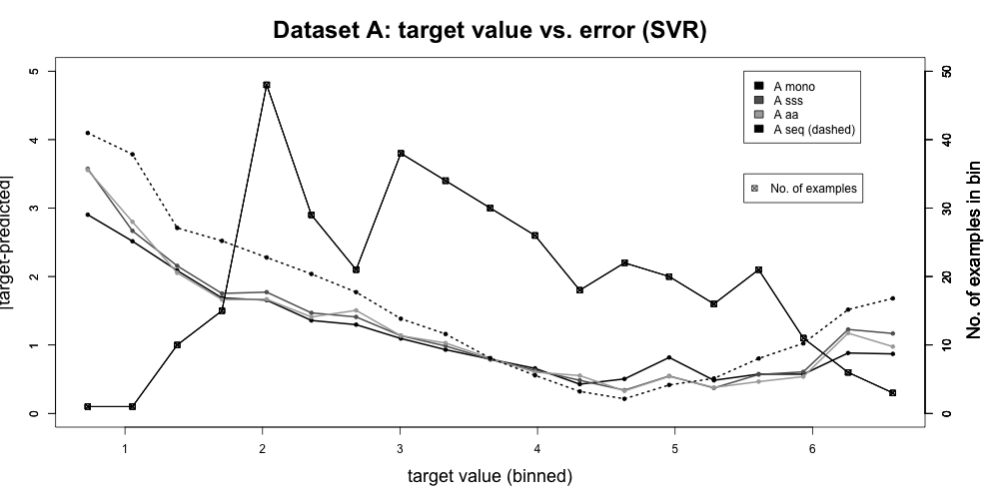
**Analysis of absolute prediction error**. Plot of target value vs. prediction error. Data was pooled into 20 bins according to their target values. For each bin, the mean absolute prediction error is plotted on the left y-axis. Then the number of values falling into the corresponding bin is shown with squares on the right y-axis. The lowest error is achieved for intermediate target values, the highest error occurs for low ones. The absolute error is not correlated to the number of values per bin. Thus, intensities within a certain range are more difficult to predict than others.

The most reliable prediction for both datasets is achieved with slightly above intermediate intensities. Low target values have the highest prediction error (Fig. 4). Note that there is a large number of samples with small target values in our training sets, so this effect cannot be attributed to undersampling, meaning that low intensities are more difficult to predict. Note that we predict the logarithm of intensities, whereas noise in the mass spectra is additive and, hence, will have a stronger effect at low intensities. Also, noise in regions of lower intensities behaves differently from that of higher intensities [21]. The problem might be overcome when more measurements for each peptide become available. Otherwise, the use of two or more different models specialized for different intensity ranges might overcome this problem.

### Dependence on the learning architecture

Of all the learning architectures *ν*-SVR gives the best results in all cases, and the LLM's performance is comparable in the cross-validation and slightly worse in the generalization case (Table 2). Where data becomes available gradually during experiments, we suggest applying the LLM in early stages: it is faster to train than the *ν*-SVR and can be adapted with additional data without time-consuming complete retraining. When enough data has been collected and results begin to stabilize, the *ν*-SVR should be used to obtain a final prediction model. The LM shows bad overfitting: It beats the LLM for the *aa *feature set in the cross-validation, but shows absolutely no correlation when generalizing to new peptides, and only very little correlation for the lower-dimensional feature sets. This suggests a non-linear relationship between feature vectors and the target values for any of the peptide representations.

### Influence of features sets

A comparison of different feature sets shows that the *mono *and *sss *feature sets generally lead to similar prediction results, the *sss *feature set having a slight advantage in more cases than the *mono *set. Thus, our feature selection increases accuracy slightly for most cases. For the 531-dimensional *aa *feature set, only the *ν*-SVR successfully extracts the relevant information to achieve comparable prediction accuracy. For the other learning algorithms, the high dimensionality with some features being highly correlated to each other, leads to bad generalization performance, i.e. inability to predict intensities for new peptides. The sparse 9220-dimensional *seq *feature set performs worse than any other feature set. While, in principle, this feature set captures more information about amino acid order, it is inappropriate for our small training datasets. This is a general problem: there are indications that not only the amino acid frequencies but also their order determine peak intensities. However, the amount of information necessary to capture this relationship explodes. Even if the amino acid order is encoded only partially, as in this case, more training data is needed.

### Analysis of the selected features

The features selected most often in the **feature selection **on dataset *A *were the estimated gas-phase basicity at 500 K (GB500), the absolute number of arginine residues (arginin_count), the relative population of conformational state E (VASM830103 [38]), the hydropathy scale based on self-information values in the two-state model at 36% accessibility (NADH010106 [39]), the hydrophobicity coefficient in RP-HPLC, C8 with 0.1%TFA/MeCN/H_2_O (WILM950102 [40], and the number of positive charges (FAUJ880111 [41]) of the *aa *feature set. From the *seq *feature set, the numbers of arginine (R), phenylalanine (F), and methionine (M) residues were selected most often. Table 1 shows the exact numbers. Details of the results of the forward stepwise selection (selected features and performance values) can be found in additional file 9: stepwise selection.

Forward stepwise selection is a greedy method and does not find an optimal solution. None of the selected sets from each single run of the method leads to better performance than that of the original *aa *set. Thus, we add in other features that extend the description of the peptide.

**Figure 5:**
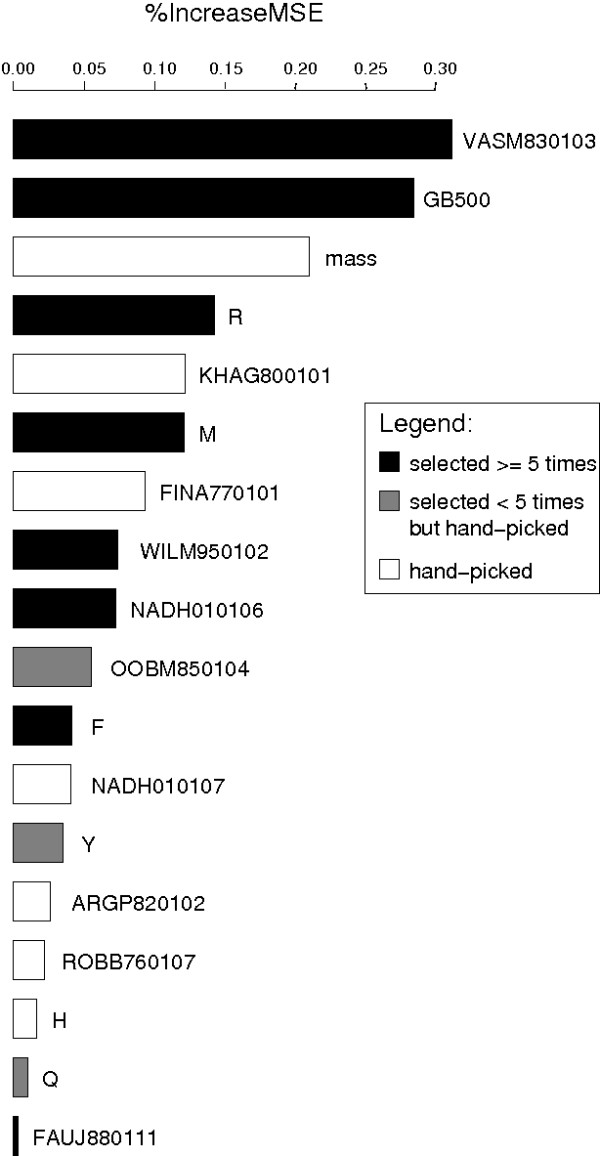
**Feature importance**. Plot of percentage increase of the prediction error if the corresponding feature is randomly permuted, using random forests for regression [42]. Of all features in the *sss *feature set, the relative population of conformational state E (VASM830103, [38]), the estimated gas-phase basicity (GB500, [36]), and the theoretical mass lead to the highest increase of the error if the peptide's values are permuted. The number of positive charges (FAUJ880111, [41]) and the number of glutamine residues (Q) are rated the least important features.

We access the importance of the single features that constitute the final *sss *feature set using random forests for regression [42,43]. Fig. 5 visualizes the percentage increase of the prediction error if values of the corresponding feature are randomly permuted. According to this, VASM830103 is the most important feature, followed by GB500 and the peptide's theoretical mass.

In general, the automatically selected features are of higher importance than hand-picked ones (Fig. 5). Of the hand-picked features, the mass and Kerr-constant increments (KHAG800101 [44]) show the highest increase in error, i.e. are most important.

The high importance of VASM830103 indicates that the amino acids' conformation might play a role for peptide-specific instrument sensitivity. However, not much is known about the conformation of peptides after tryptic digestion, when crystallized within the matrix, or as ions in gas-phase. Looking at the chemistry, it makes sense that the gas-phase basicity influences ionization efficiency. The number of positive charges have been reported by Mallick *et al*. to be relevant for the probability of observing a peptide ion [14]. The latter has often been chosen in the feature selection but is the least important one in the *sss *feature set according to our feature importance accession. The number of histidine residues (H) has been found to be correlated with detection probabilities in MALDI MS by Mallick *et al*.. It is the only basic residue except for K and R, presumably making a difference, though weakly, in basicity for tryptic, i.e. already quite basic peptides. However, it is one of the three least important features according to the random forest method in our dataset. We can exclude the correlation (*r *= 0.922) between H and FAUJ880111 as the cause of their low importance: the importance ranking is the same if one of both is left out completely. The hydrophobicity features (WILM950102, NADH010106) are of intermediate importance. Since all except a few peptides in the studied datasets are tryptic, i.e. have lysine (K) or arginine (R) as the C-terminal amino acid, the R feature value almost always indicates whether the peptide string ends in R or not. In the latter case, it most certainly ends in K. The feature R is highly correlated with the GB500 value: the estimated gas-phase basicity takes on values distributed around three levels, which correspond to peptides ending in R (highest), those ending in K, and those that have neither. Nonetheless, both features seem to be complementary according to our results.

A **two-sample t-test **of the list of normalized intensities of peptides *containing *a given mono- or di-, or tri-peptide substring against the list of those *not containing *it can show whether differences in the mean intensities between these groups are significant. We apply this test to complement the information obtained by the forward stepwise selection on *seq*. In this test, each substring is tested independently of all others, whereas the forward selection would not choose a substring if its effect is already covered by another one. Details including *p*-values are given in Table 4. The amino acid features number of arginine (R), lysine (K), methionine (M), and the terminal di-peptide GK show significant differences between their mean values for both datasets. Apart from GK, significant di-peptides differ between both datasets and almost always contain one of the aforementioned (R, K, M), so their effect can be attributed to these single amino acids. In dataset *A*, there are significant differences for histidine (H), tyrosine (Y), phenylalanine (F), and glutamine (Q), whereas none of these shows up in dataset *B*. Here, tryptophane (W) containing peptides show a significant difference in the mean intensity instead. If unmatched peptides are included with a target value of zero, the amino acids threonine (T) and tryptophan (W) show a significant difference for both datasets (data not shown).

**Table 4:** Two-sample t-test results

**substring *s***	**p-value**	*μ*($s_{A}^{-}$)	*μ*($s_{A}^{+}$)	**size**($s_{A}^{-}$)	**size**($s_{A}^{+}$)
**R**	**2.41e-15**	**2.76**	**3.73**	**171**	**244**
**K**	**2.85e-14**	**3.72**	**2.78**	**243**	**172**
**M**	**1.69e-06**	**3.44**	**2.53**	**366**	**49**
H	2.25e-05	3.20	3.90	338	77
VKe	4.64e-05	3.36	2.31	403	12
VK	8.92e-05	3.36	2.39	402	13
VF	1.25e-04	3.29	4.65	403	12
Y	2.11e-04	3.18	3.71	296	119
F	2.99e-04	3.15	3.67	272	143
GF	5.83e-04	3.29	4.48	400	15
Q	8.97e-04	3.16	3.61	260	155
**GKe**	**0.001**	**3.37**	**2.62**	**392**	**23**
TKe	0.001	3.36	2.45	401	14
SV	0.001	3.28	4.15	393	22
TK	0.003	3.36	2.53	400	15
GK	0.009	3.37	2.76	390	25
PR	0.009	3.30	4.51	403	12
PRe	0.009	3.30	4.51	403	12
DS	0.009	3.29	4.10	395	20

**substring *s***	**p-value**	*μ*($s_{B}^{-}$)	*μ*($s_{B}^{+}$)	**size**($s_{B}^{-}$)	**size**($s_{B}^{+}$)

**R**	**4.12e-33**	**3.69**	**4.64**	**339**	**795**
**K**	**6.76e-31**	**4.65**	**3.74**	**770**	**364**
**M**	**3.72e-25**	**4.52**	**3.41**	**966**	**168**
DK	3.80e-07	4.38	3.35	1112	22
DKe	1.18e-06	4.37	3.38	1113	21
GM	1.67e-05	4.38	3.23	1112	22
AKe	2.27e-05	4.39	3.64	1085	49
NKe	5.82e-05	4.38	3.42	1111	23
GRe	9.18e-05	4.31	4.93	1054	80
QRe	1.37e-04	4.33	5.10	1100	34
W	2.63e-04	4.40	3.93	1034	100
AK	2.75e-04	4.39	3.73	1083	51
NK	3.03e-04	4.37	3.51	1110	24
GR	6.16e-04	4.32	4.86	1051	83
QR	7.46e-04	4.34	5.01	1098	36
FRe	0.001	4.34	5.28	1111	23
IK	0.001	4.37	3.47	1113	21
IKe	0.001	4.37	3.47	1113	21
**GKe**	**0.001**	**4.37**	**3.74**	**1104**	**30**
GK	0.002	4.37	3.80	1101	33
DT	0.003	4.38	3.80	1083	51
AM	0.003	4.38	3.40	1108	26
S	0.004	4.47	4.25	538	596
P	0.006	4.25	4.46	579	555
TKe	0.008	4.37	3.76	1110	24
VRe	0.008	4.33	4.77	1069	65

Results of two-sample t-tests of set *s*^+ ^(normalized intensities of peptides containing a substring s) against the set *s*^- ^of those not containing it in the corresponding dataset (*A *or *B*). Only substrings that occur in more than 10 (*A*)/20 (*B*) peptides and with a p-value ≤ 0.001 are shown. An "a" as a prefix denotes that the substring is located at the beginning of the string, "e" as suffix means it is located at the end of the string. Otherwise, the substring can occur anywhere in the peptide (including terminal positions). Rows in bold face mark substrings that are present in the lists of both datasets.

### Dataset dependence

Generally, dataset *A *gives much better results than *B*, even though the latter is larger (see Table 2 as well as additional files of *A *and *B *scatter plots referred to in Table 3). An obvious reason is the higher within-peptide variance of normalized intensities and the higher fraction of peptides without replicate measurements in dataset *B*. Nonetheless, the algorithms are able to draw the main trends from *B*, since *A *can be predicted with a model trained on *B *even better than *B *itself (compare "across datasets" for *A *to "10-fold CV" for *B *in Table 2). This shows that despite the high variance in a dataset, the learning algorithms can capture the main trends, thus enabling prediction of a less noisy dataset.

## Conclusion

The machine learning approach presented here is able to predict peptide peak intensities in MALDI mass spectra, thus showing that the prediction of sensitivity factors for mass spectrometry based on chemical and sequence features is feasible. Even for small datasets, significant correlations can be achieved.

Although we have not yet evaluated our method against uncorrected spectral counts and correction based on detection probabilities, we believe that our method is better suited for peptide quantification than the latter, since we directly aim at predicting the reciprocal of the peptide-specific instrument sensitivity. As the next step in our research, we want to assess how predicted intensities affect quantification accuracy. We will apply our method to protein mixtures of known concentrations to estimate how well we can predict protein concentrations at the semi-quantitative level. Also, the application of the method to other ionization techniques, in particular data from electrospray ionization (LC-ESI), is an obvious next step. We are confident that we can improve prediction performance using larger datasets with more replicates. Regarding additional features to be included for ML, features comprising conformational aspects of peptides appear very promising. Due to the high computational costs, it will have to be assessed whether the estimation of such features is feasible for this application.

## Authors' contributions

WT generated the features, produced and evaluated the *ν*-SVR results, and did the forward feature selection. AS carried out the LLM experiments. OK contributed to the feature extraction. AS, OK, SB, and WT wrote the manuscript. TN revised and edited the manuscript. All authors agreed on the final manuscript.

## Supplementary Material

Additional file 1 **Overview of dataset properties.***No. duplicated proteins*: number of proteins for which more than one spectrum is contained (detailed numbers in parentheses). *No. matches*: number of distinct peptides for which peaks are found in the spectra, considering only peptides without missed cleavages. *No. non-matches*: number of theoretical peptides for which no match was found. *Duplicated peptides*: percentage of peptides found in more than one spectrum. *Modifications*: peptide modifications considered in the peak matching procedure. Click here for file

Additional file 2 **Histogram of the number of spectra per protein.** More than 50% of the proteins in dataset *B *are presented by only one measurement. Click here for file

Additional file 3 **Lists of the identified proteins.** Identified proteins, their coding region, description, MASCOT score, and GenDB [45] ID. Click here for file

Additional file 4 **Lists of the identified proteins.** Identified proteins, their coding region, description, MASCOT score, and GenDB [45] ID. Additional information about the MASCOT search parameters that led to the highest score are included: coverage, number of peptides (found and overall), missed cleavages, mass tolerance, modifications, and the number of search parameter sets that led to the identification of the corresponding protein. Click here for file

Additional file 5 **Raw spectra preprocessing and peak extraction details.** Click here for file

Additional file 6 **Q-Q plots for target values of both datasets.** Intensities have been normalized by *mic *and logarithmized. The target values of dataset *B *fit the normal distribution almost perfectly. Those of dataset *A *deviate from a normal distribution at both ends. Click here for file

Additional file 7 **Number of times di-/tri-peptides occur in the *seq *feature set of dataset *A*.** While a good portion of the dimers occurs more often than ten times in the whole dataset, most of the trimers do not show up at all or just once. In principle, the sequence feature set captures some of the amino acid order in the peptides. However, considerably more data is necessary to fill this feature space. Click here for file

Additional file 8 **Scatter plots of duplicate normalized intensity values against trimmed-mean target values.** The recorded correlations can be considered upper bounds of the achievable prediction performance if only multiple measurements per peptide were used (*left*: dataset *A*, *right*: dataset *B*). Click here for file

Additional file 10 **Cross-validation scatter plots and Pearson correlations for dataset *A *(*ν*-SVR, *sum *normalization).** Cross-validation scatter plots of dataset *A *with the *ν*-SVR (*sum *normalization) Click here for file

Additional file 11 **Cross-validation scatter plots and Pearson correlations for dataset *A *(LLM).** Cross-validation scatter plots of dataset *A *with the LLM (normalization: *left*: *mic*, *right*: *sum*) Click here for file

Additional file 12 **Cross-validation scatter plots and Pearson correlations for dataset *A *(*ν*-SVR, *mic *normalization).** Click here for file

Additional file 13 **Cross-validation scatter plots and Pearson correlations for dataset *B *(*ν*-SVR, *sum *normalization).** Cross-validation scatter plots of dataset *B *with the SVR (*sum *normalization) Click here for file

Additional file 14 **Cross-validation scatter plots and Pearson correlations for dataset *B *(LLM).** Cross-validation scatter plots of dataset *B *with the LLM. (normalization: *left*: *mic*, *right*: *sum*) Click here for file

Additional file 15 **Cross-validation scatter plots and Pearson correlations for dataset *B *(*ν*-SVR, *mic *normalization).** Click here for file

Additional file 16 **Across-dataset prediction scatter plots and Pearson correlations for dataset *A *(*ν*-SVR *sum *normalization).** Across-dataset prediction of dataset *A *with a model from *B *with the *ν*-SVR (*sum *normalization) Click here for file

Additional file 17 **Across-dataset prediction scatter plots and Pearson correlations for dataset *A *(LLM).** Across-dataset prediction of dataset *A *with a model from *B *with the LLM (normalization: *left*: *mic*, *right*: *sum*) Click here for file

Additional file 18 **Across-dataset prediction scatter plots and Pearson correlations for dataset *A *(*ν*-SVR, *mic *normalization).** Click here for file

Additional file 19 **Across-dataset prediction scatter plots and Pearson correlations for dataset *B *(*ν*-SVR, *sum *normalization).** Across-dataset prediction of dataset *B *with a model from *A *with the *ν*-SVR (*sum *normalization) Click here for file

Additional file 20 **Across-dataset prediction scatter plots and Pearson correlations for dataset *B *(LLM).** Across-dataset prediction of dataset *B *with a model from *A *with the LLM (normalization: *left*: *mic*, *right*: *sum*) Click here for file

Additional file 21 **Across-dataset prediction scatter plots and Pearson correlations for dataset *B *(*ν*-SVR, *mic *normalization).** Click here for file

Additional file 9 **Details of the forward stepwise selection process.** Features selected as well as performance values for each run of the forward stepwise selection process are shown in separate tables for selection in *aa *and *seq*. Click here for file
