# Supplementary figures and images for: Peak intensity prediction in MALDI-TOF mass spectrometry: A machine learning study to support quantitative proteomics
Source: BMC Bioinformatics. 2008 Oct 20;9:443. doi: 10.1186/1471-2105-9-443 (PMC2600826; doi:10.1186/1471-2105-9-443)

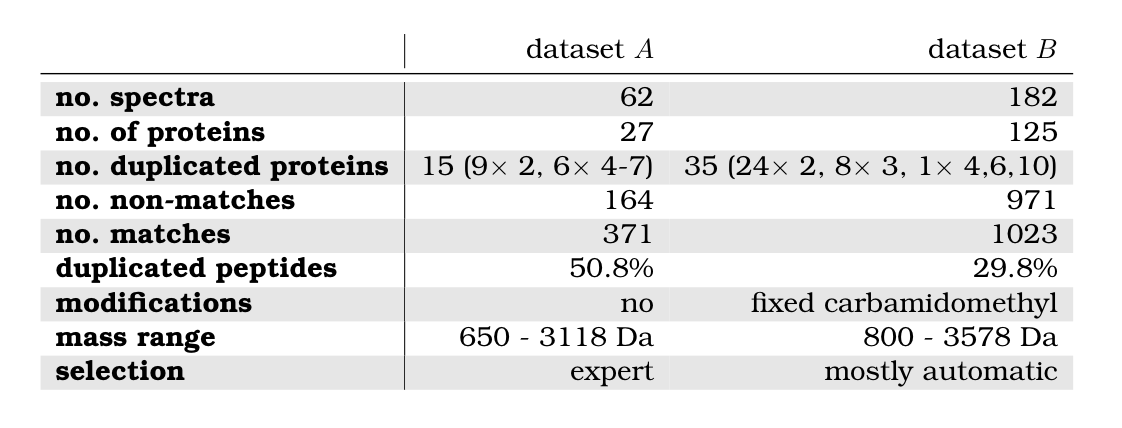

Supplement: Additional file 1 — Overview of dataset properties.No. duplicated proteins: number of proteins for which more than one spectrum is contained (detailed numbers in parentheses). No. matches: number of distinct peptides for which peaks are found in the spectra, considering only peptides without missed cleavages. No. non-matches: number of theoretical peptides for which no match was found. Duplicated peptides: percentage of peptides found in more than one spectrum. Modifications: peptide modifications considered in the peak matching procedure. [file 1471-2105-9-443-S1.png]

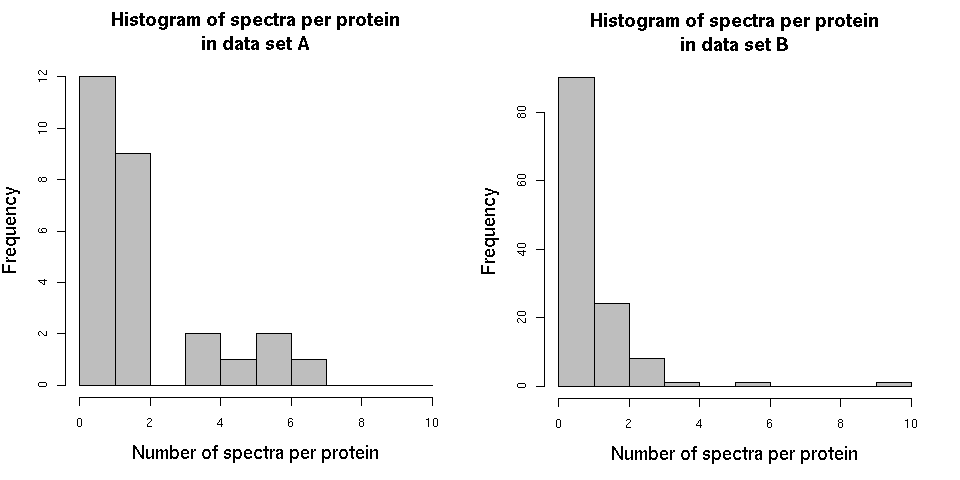

Supplement: Additional file 2 — Histogram of the number of spectra per protein. More than 50% of the proteins in dataset B are presented by only one measurement. [file 1471-2105-9-443-S2.png]

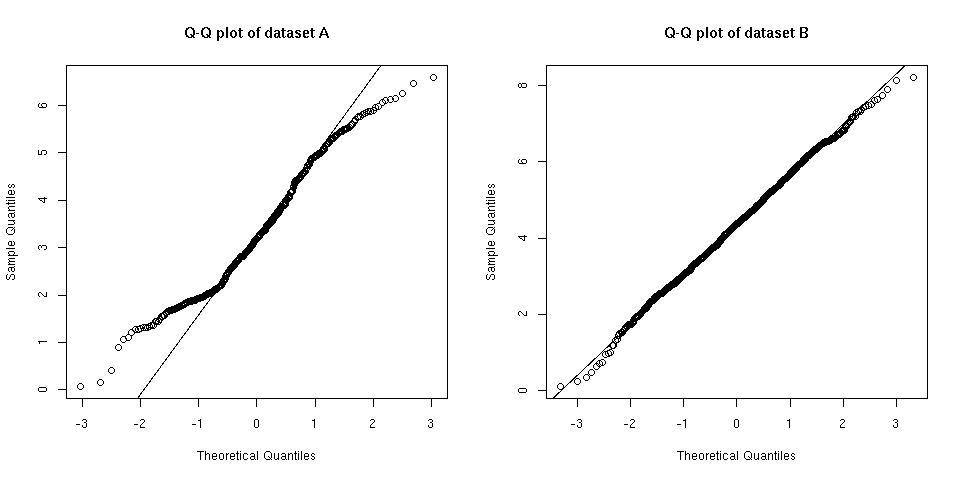

Supplement: Additional file 6 — Q-Q plots for target values of both datasets. Intensities have been normalized by mic and logarithmized. The target values of dataset B fit the normal distribution almost perfectly. Those of dataset A deviate from a normal distribution at both ends. [file 1471-2105-9-443-S6.png]

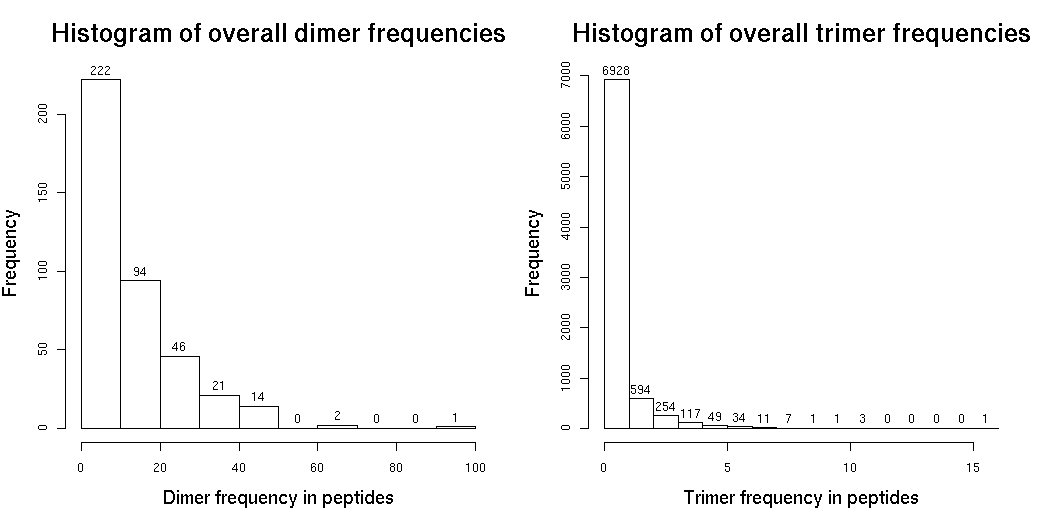

Supplement: Additional file 7 — Number of times di-/tri-peptides occur in the seq feature set of dataset A. While a good portion of the dimers occurs more often than ten times in the whole dataset, most of the trimers do not show up at all or just once. In principle, the sequence feature set captures some of the amino acid order in the peptides. However, considerably more data is necessary to fill this feature space. [file 1471-2105-9-443-S7.png]

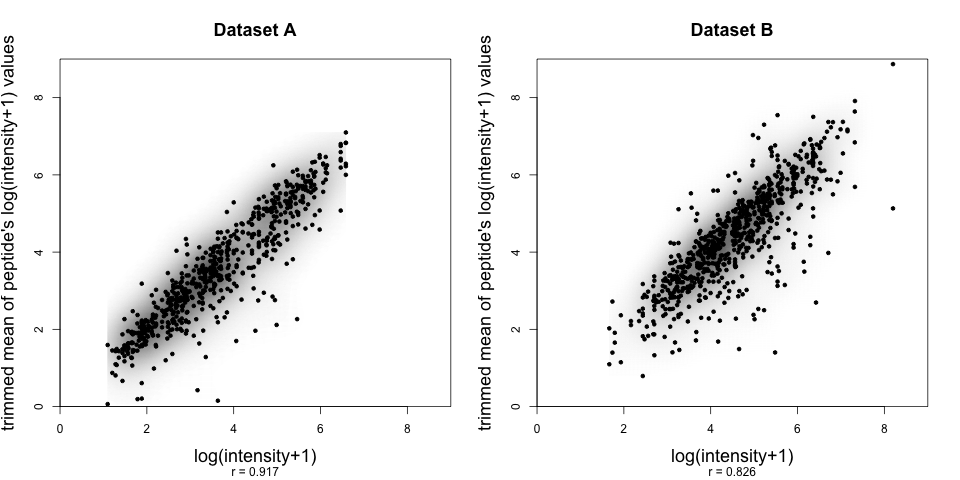

Supplement: Additional file 8 — Scatter plots of duplicate normalized intensity values against trimmed-mean target values. The recorded correlations can be considered upper bounds of the achievable prediction performance if only multiple measurements per peptide were used (left: dataset A, right: dataset B). [file 1471-2105-9-443-S8.png]

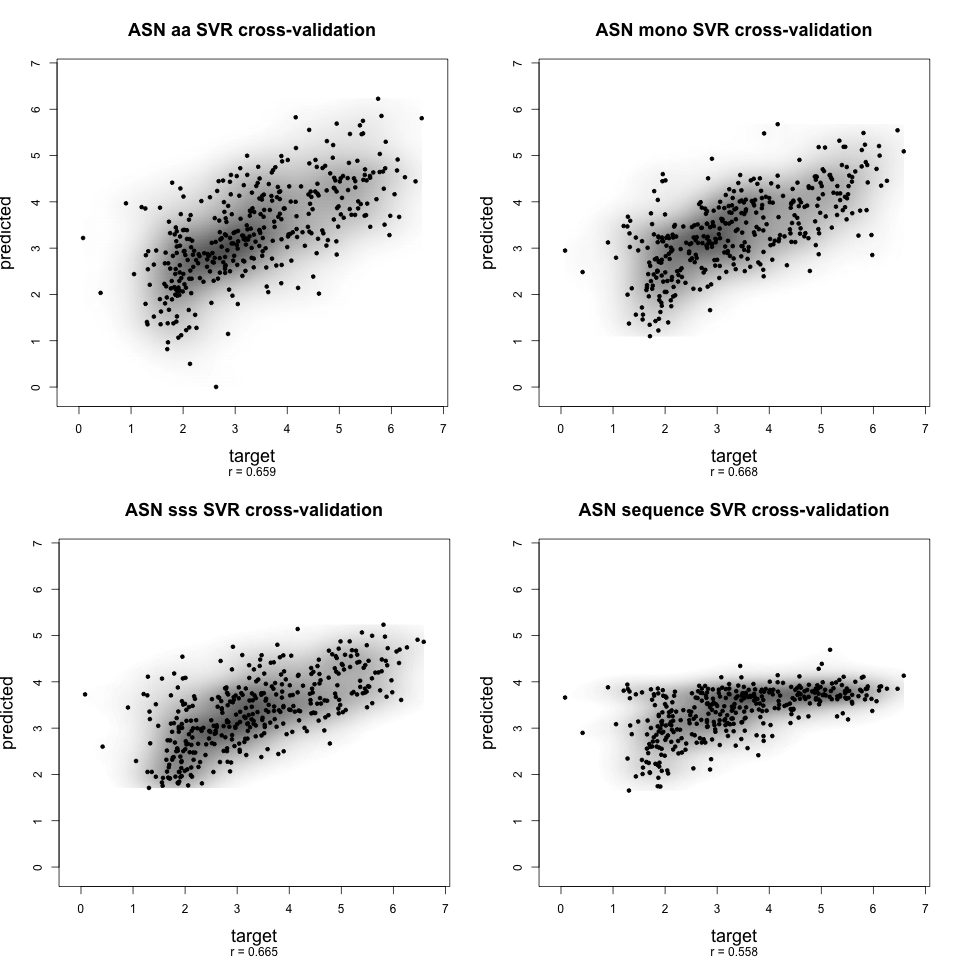

Supplement: Additional file 10 — Cross-validation scatter plots and Pearson correlations for dataset A (ν-SVR, sum normalization). Cross-validation scatter plots of dataset A with the ν-SVR (sum normalization) [file 1471-2105-9-443-S10.png]

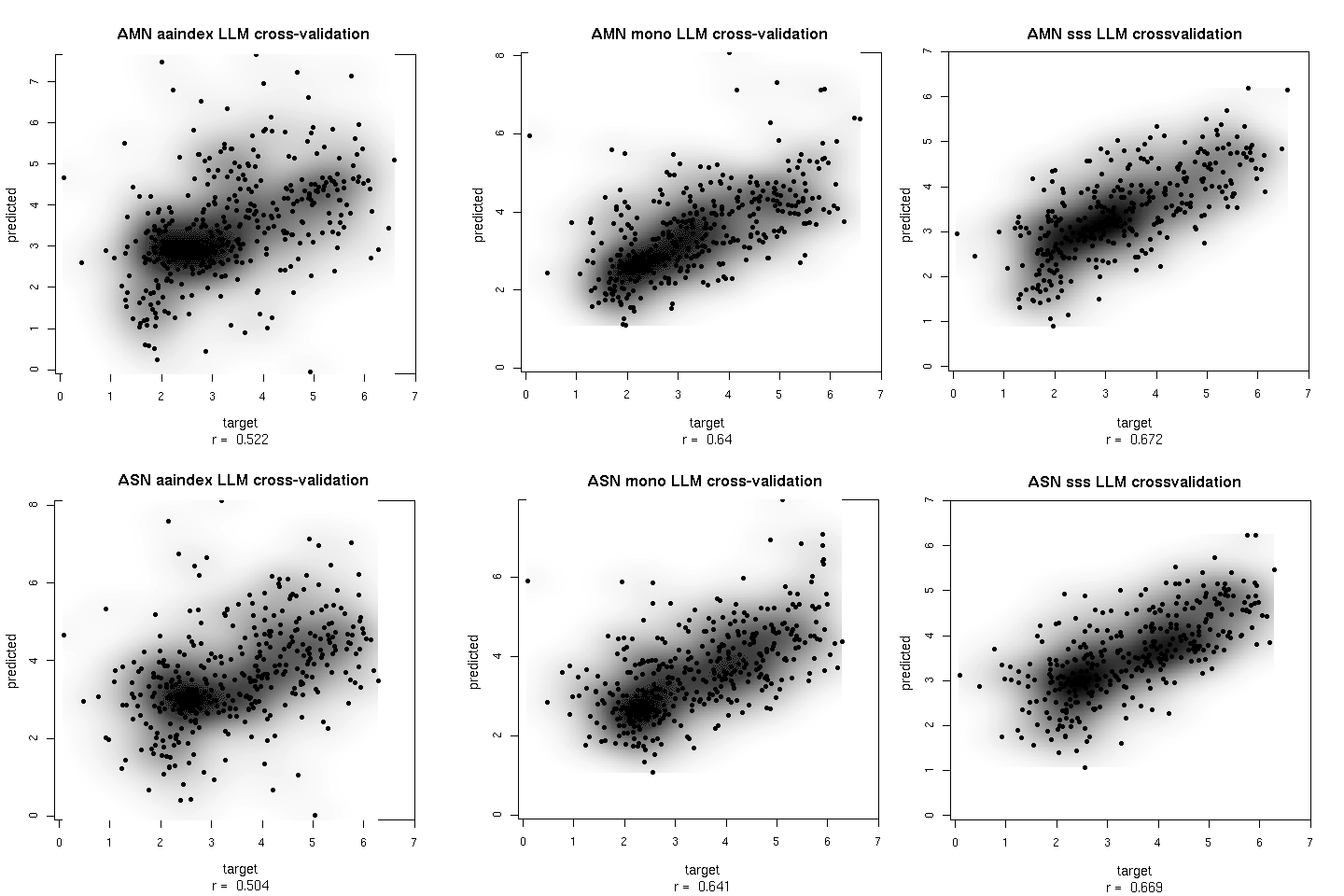

Supplement: Additional file 11 — Cross-validation scatter plots and Pearson correlations for dataset A (LLM). Cross-validation scatter plots of dataset A with the LLM (normalization: left: mic, right: sum) [file 1471-2105-9-443-S11.png]

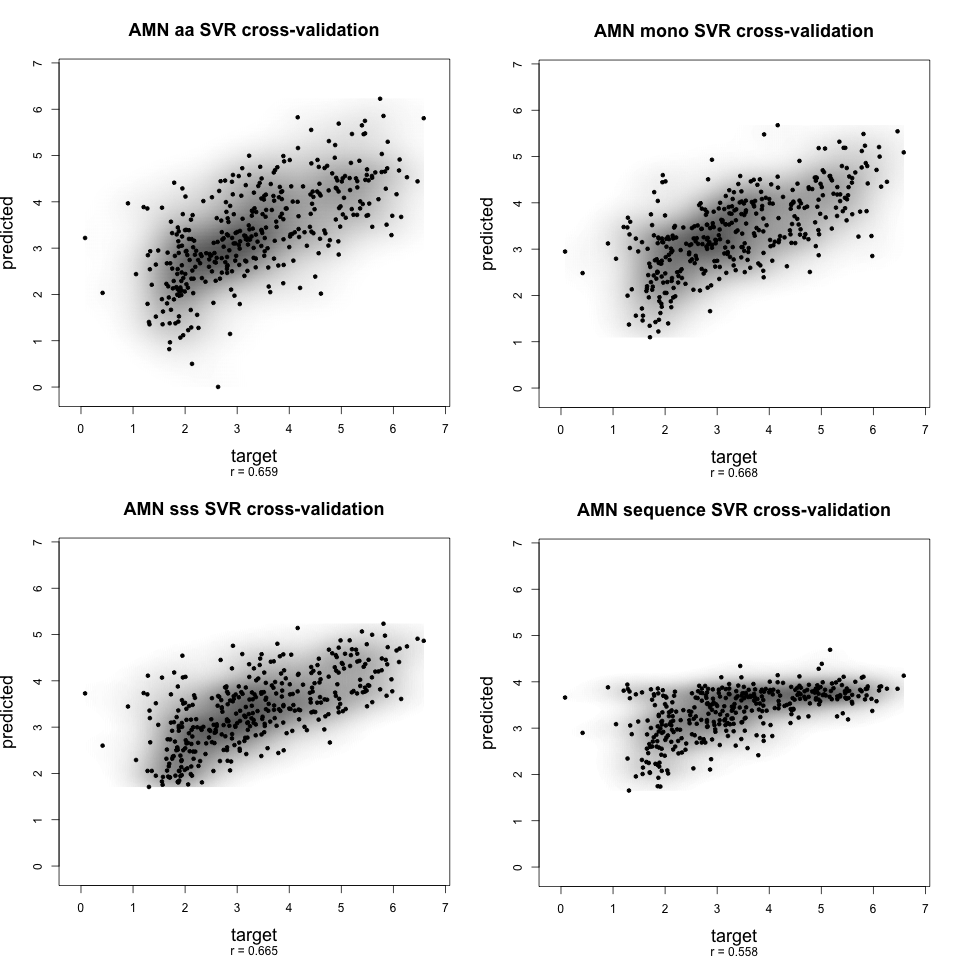

Supplement: Additional file 12 — Cross-validation scatter plots and Pearson correlations for dataset A (ν-SVR, mic normalization). [file 1471-2105-9-443-S12.png]

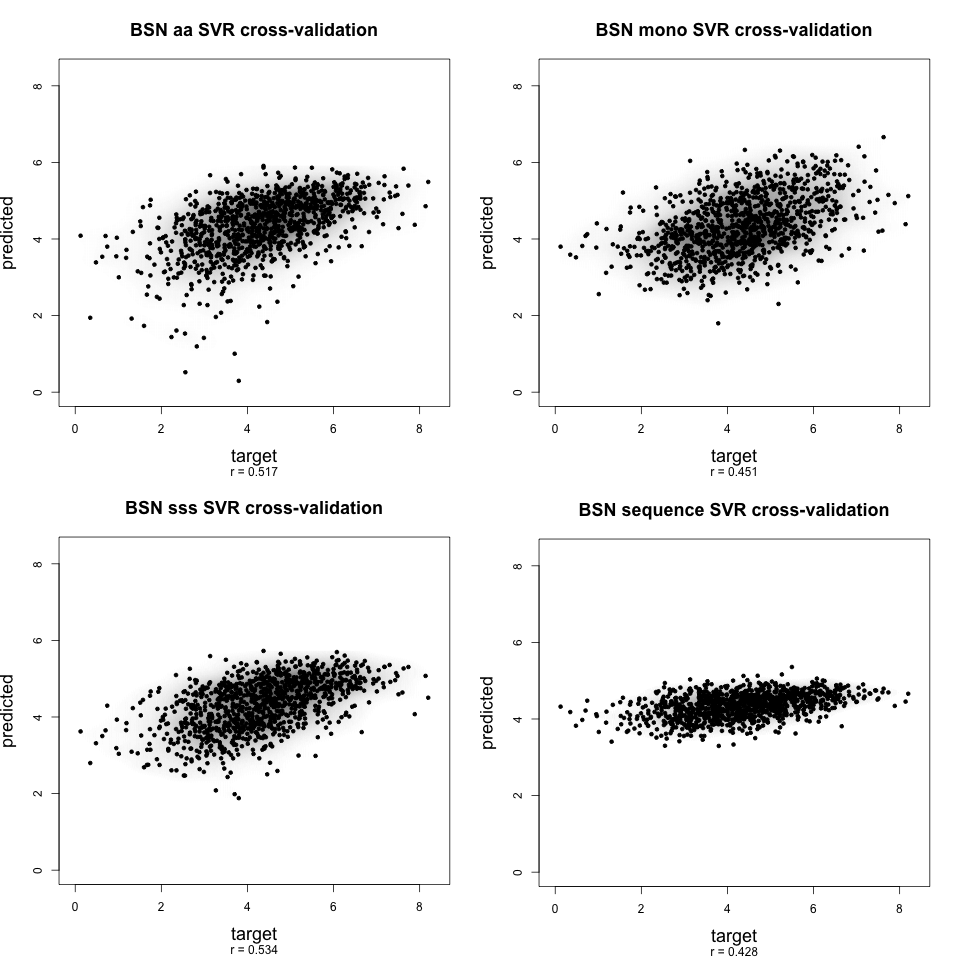

Supplement: Additional file 13 — Cross-validation scatter plots and Pearson correlations for dataset B (ν-SVR, sum normalization). Cross-validation scatter plots of dataset B with the SVR (sum normalization) [file 1471-2105-9-443-S13.png]

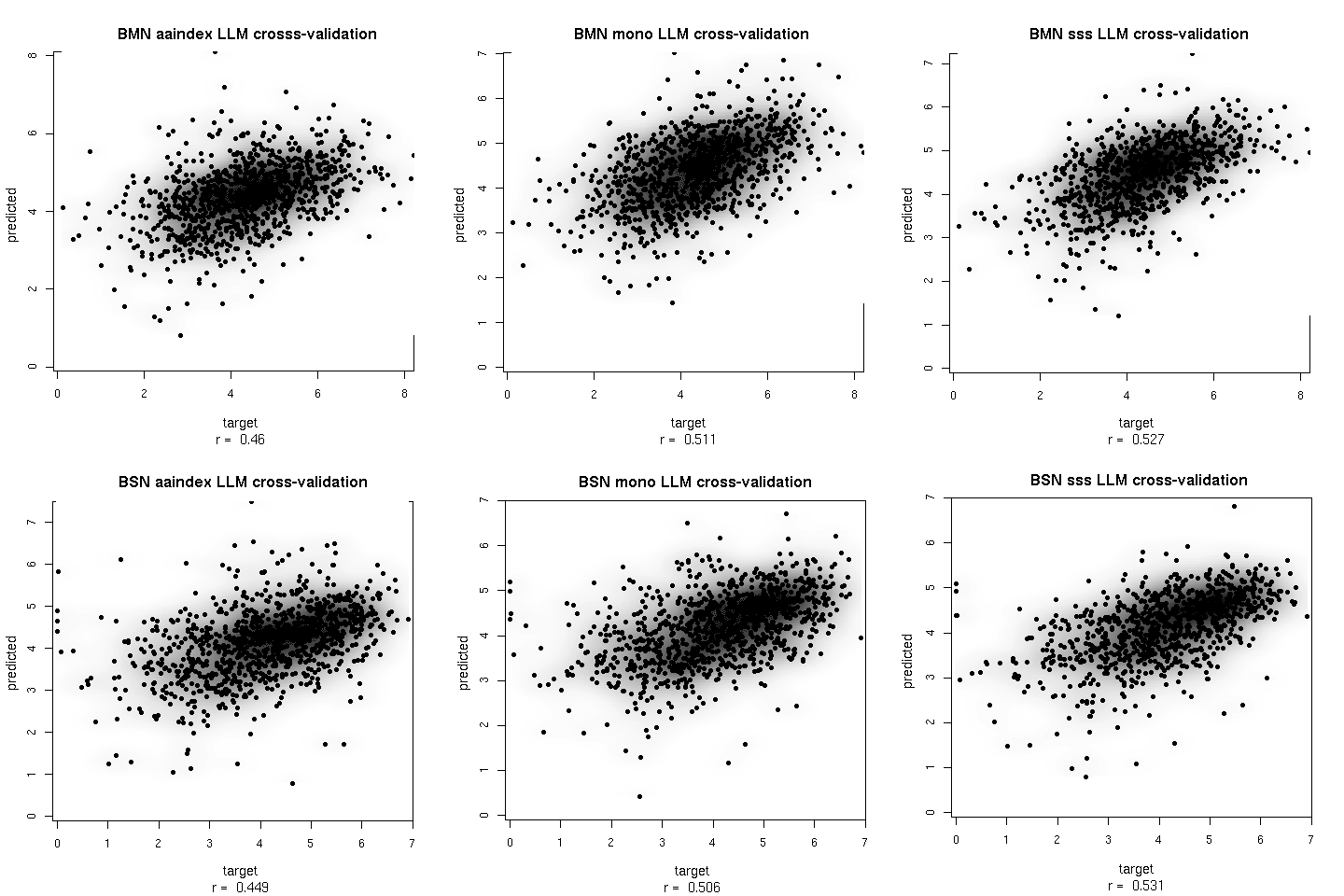

Supplement: Additional file 14 — Cross-validation scatter plots and Pearson correlations for dataset B (LLM). Cross-validation scatter plots of dataset B with the LLM. (normalization: left: mic, right: sum) [file 1471-2105-9-443-S14.png]

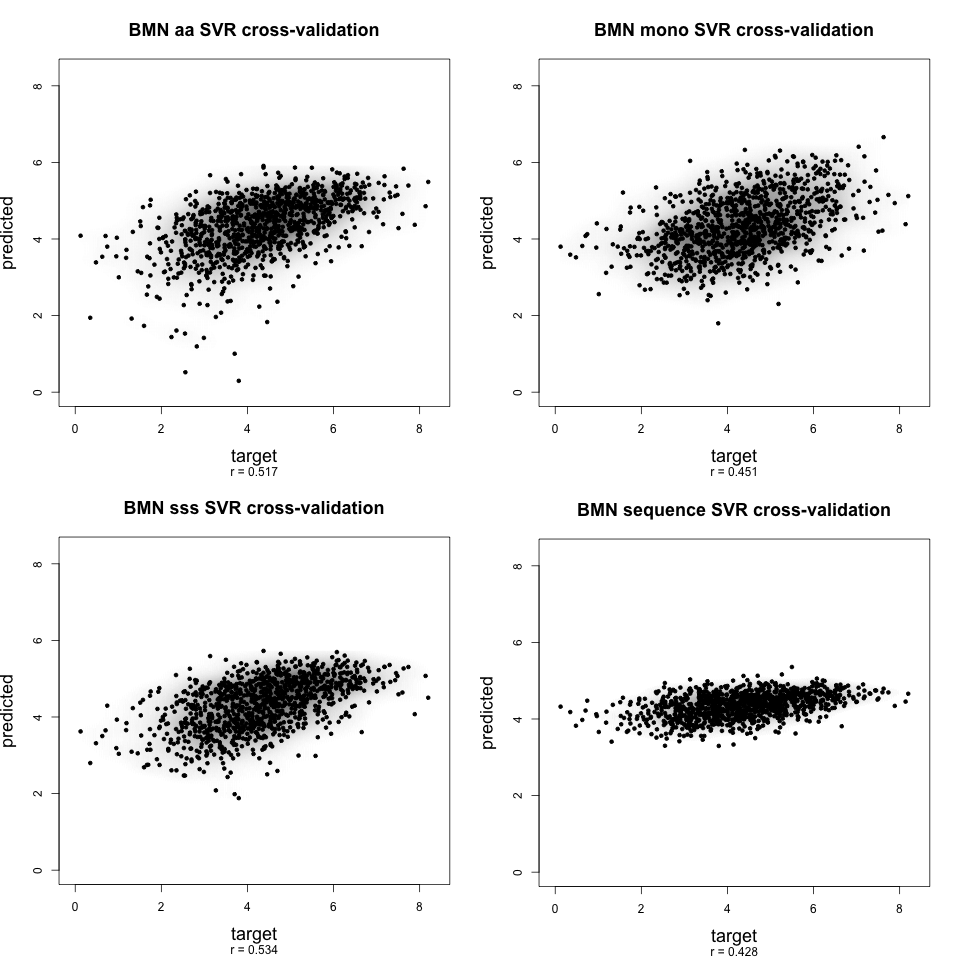

Supplement: Additional file 15 — Cross-validation scatter plots and Pearson correlations for dataset B (ν-SVR, mic normalization). [file 1471-2105-9-443-S15.png]

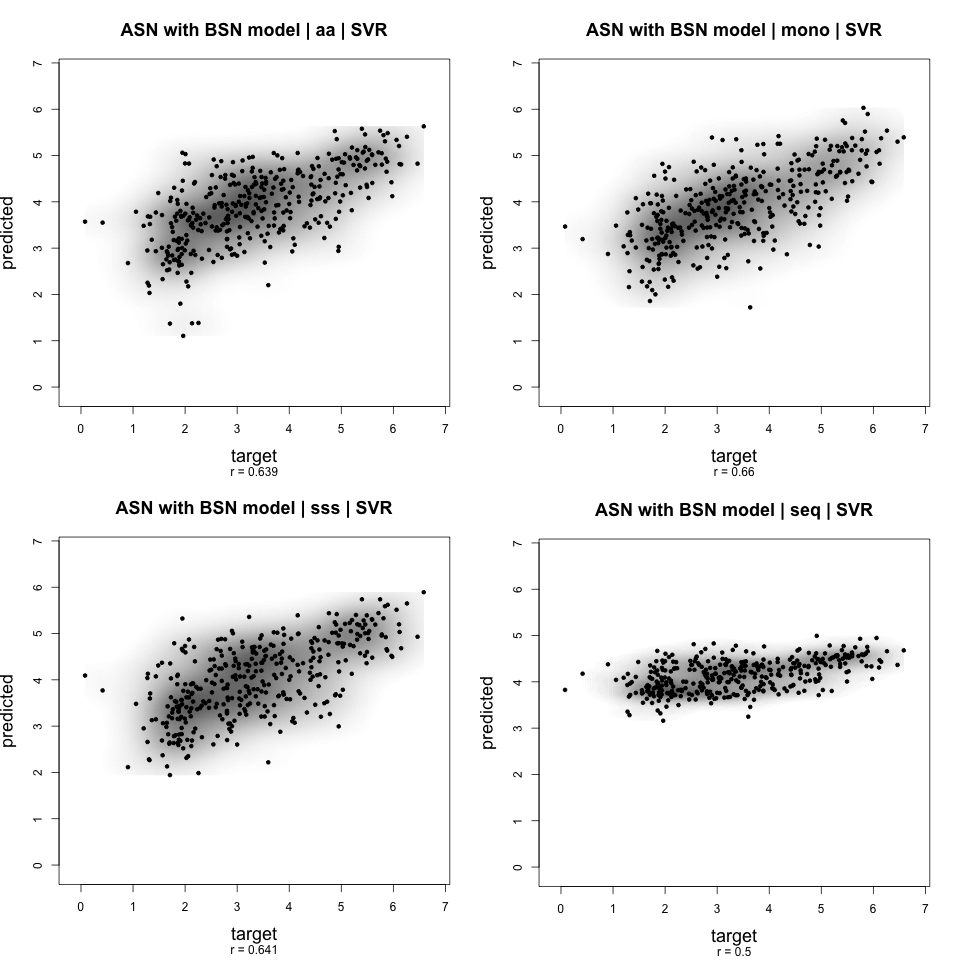

Supplement: Additional file 16 — Across-dataset prediction scatter plots and Pearson correlations for dataset A (ν-SVR sum normalization). Across-dataset prediction of dataset A with a model from B with the ν-SVR (sum normalization) [file 1471-2105-9-443-S16.png]

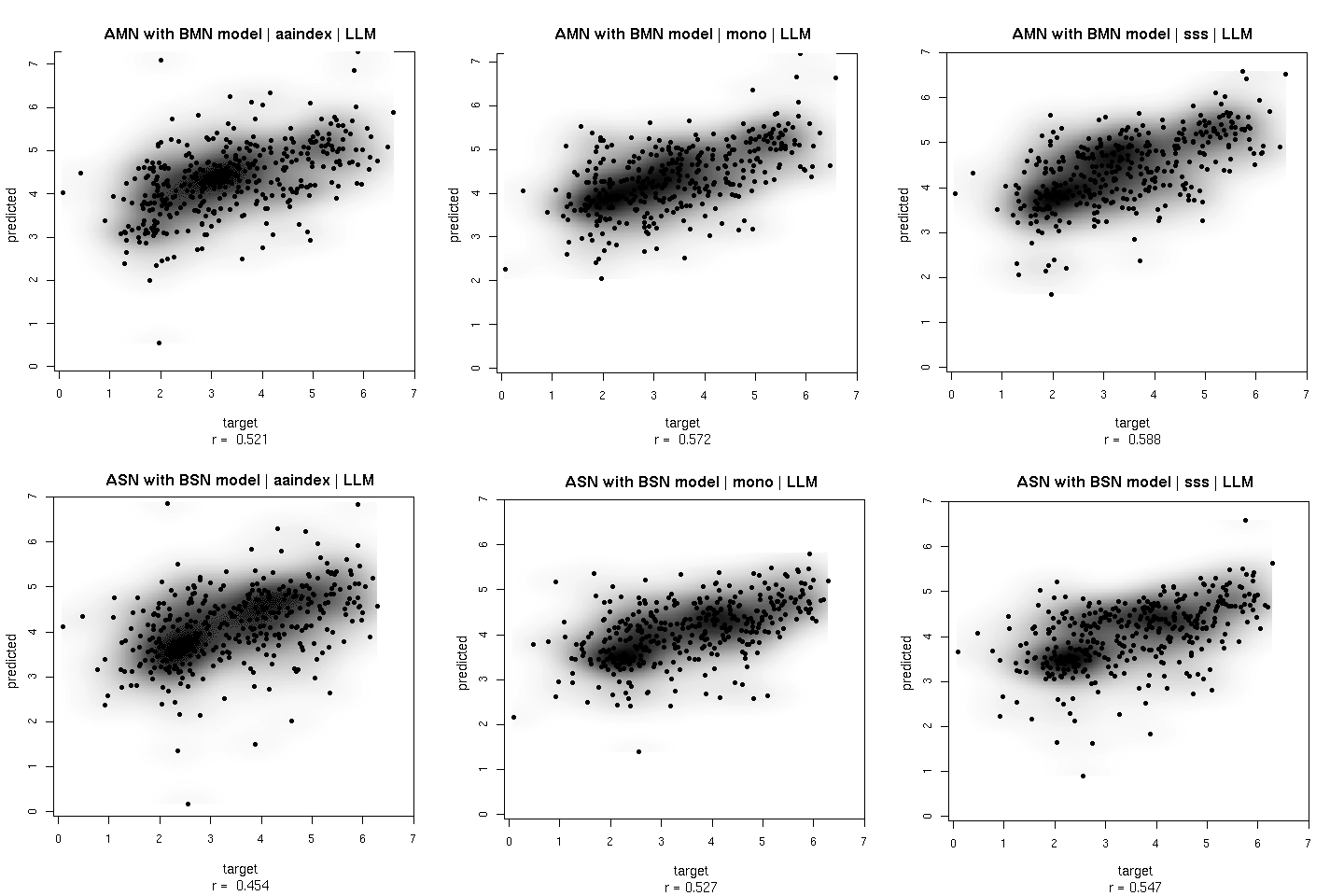

Supplement: Additional file 17 — Across-dataset prediction scatter plots and Pearson correlations for dataset A (LLM). Across-dataset prediction of dataset A with a model from B with the LLM (normalization: left: mic, right: sum) [file 1471-2105-9-443-S17.png]

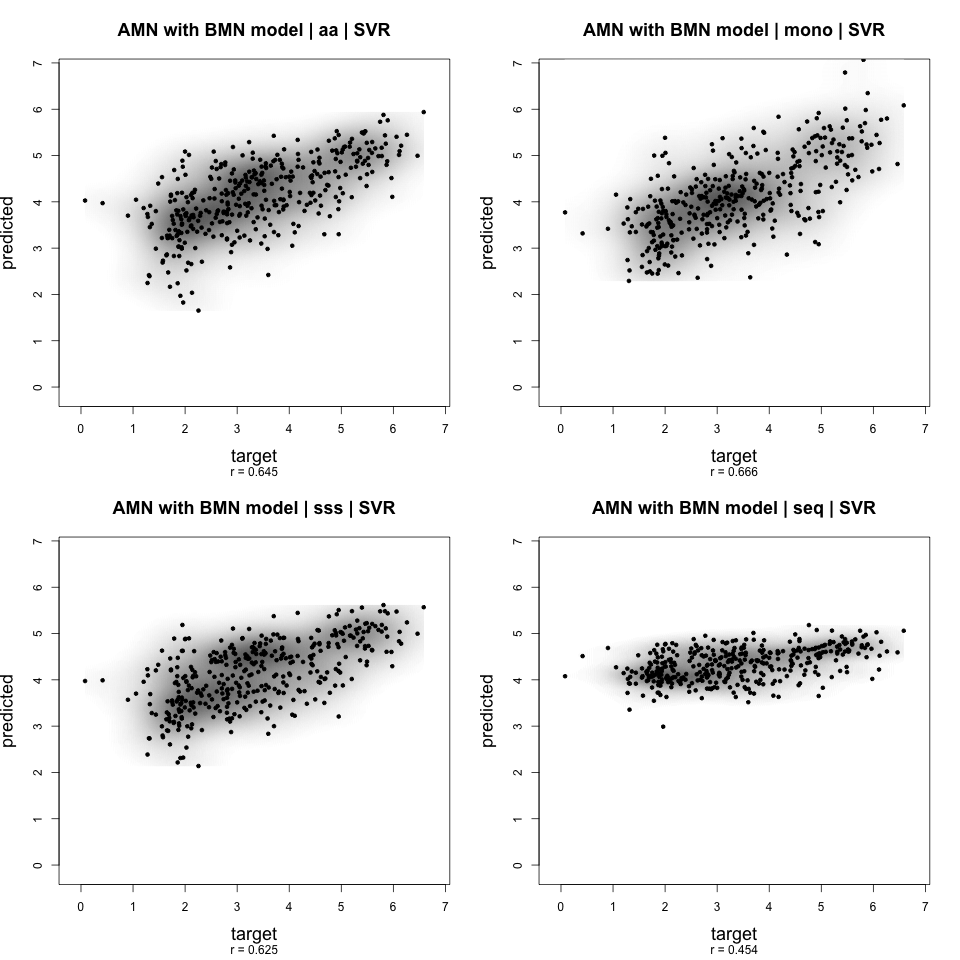

Supplement: Additional file 18 — Across-dataset prediction scatter plots and Pearson correlations for dataset A (ν-SVR, mic normalization). [file 1471-2105-9-443-S18.png]

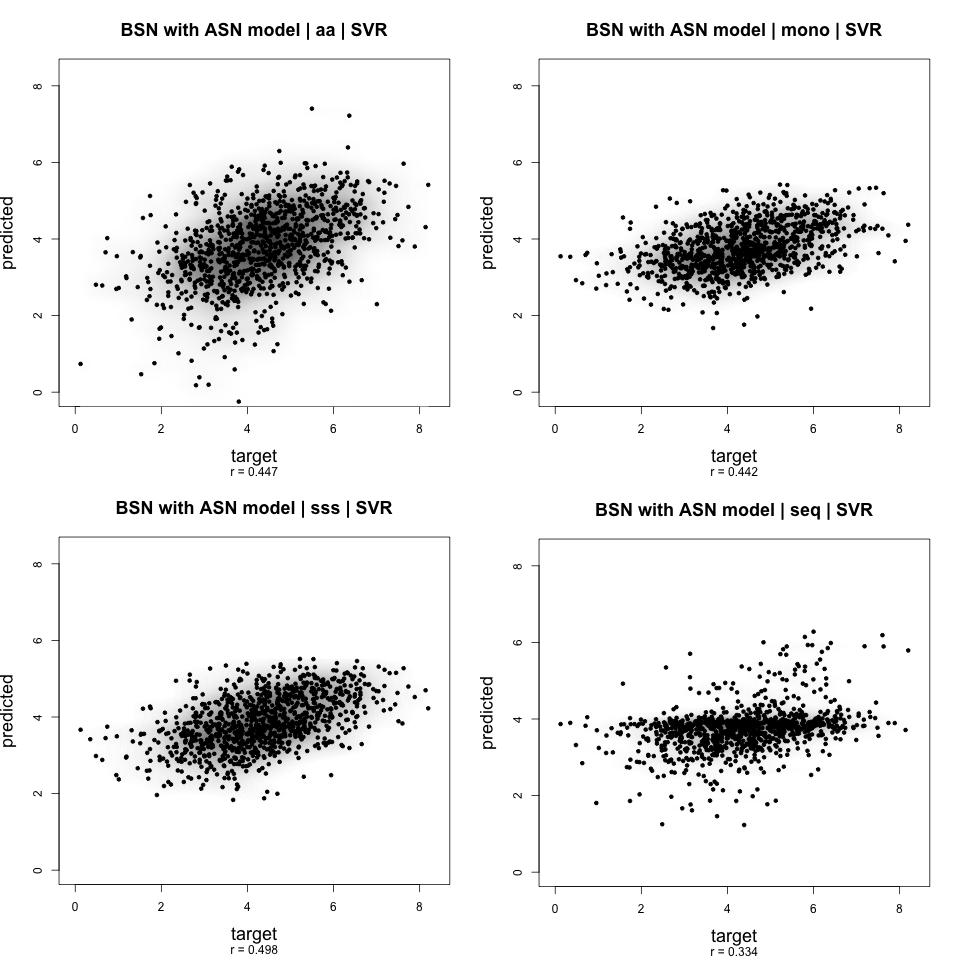

Supplement: Additional file 19 — Across-dataset prediction scatter plots and Pearson correlations for dataset B (ν-SVR, sum normalization). Across-dataset prediction of dataset B with a model from A with the ν-SVR (sum normalization) [file 1471-2105-9-443-S19.png]

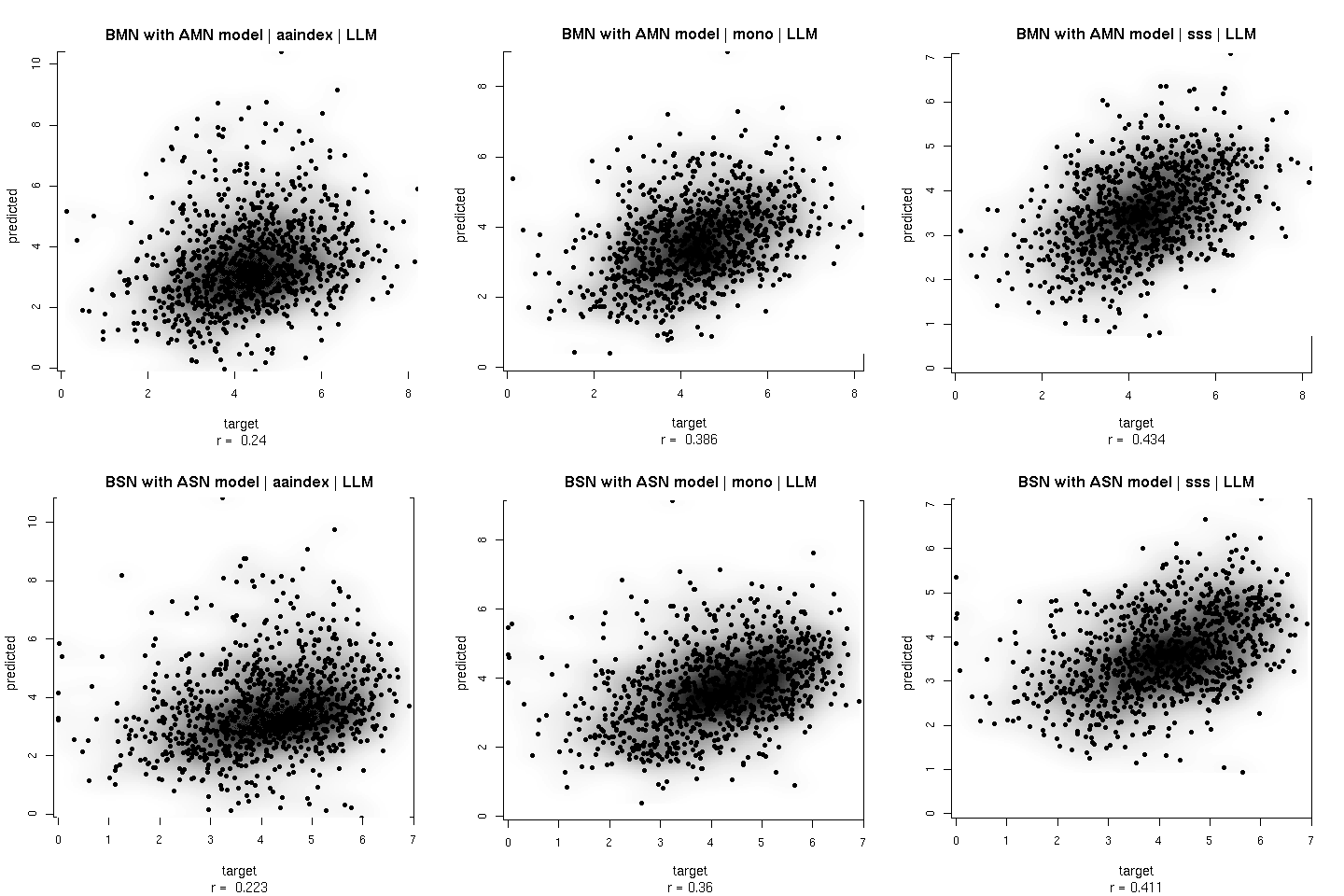

Supplement: Additional file 20 — Across-dataset prediction scatter plots and Pearson correlations for dataset B (LLM). Across-dataset prediction of dataset B with a model from A with the LLM (normalization: left: mic, right: sum) [file 1471-2105-9-443-S20.png]

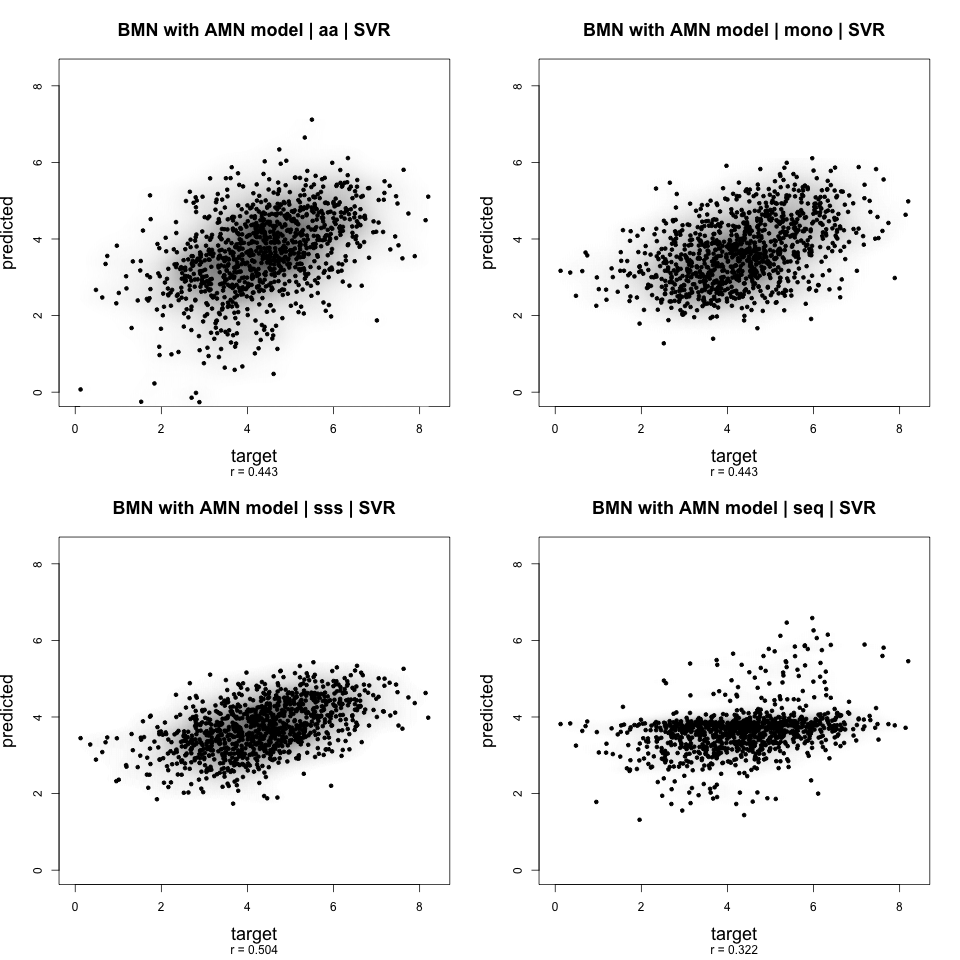

Supplement: Additional file 21 — Across-dataset prediction scatter plots and Pearson correlations for dataset B (ν-SVR, mic normalization). [file 1471-2105-9-443-S21.png]
