# Supplementary material for: Peak intensity prediction in MALDI-TOF mass spectrometry: A machine learning study to support quantitative proteomics
Source: BMC Bioinformatics. 2008 Oct 20;9:443. doi: 10.1186/1471-2105-9-443 (PMC2600826; doi:10.1186/1471-2105-9-443)
Supplement: Additional file 3 — Lists of the identified proteins. Identified proteins, their coding region, description, MASCOT score, and GenDB [45] ID. [file 1471-2105-9-443-S3.pdf]

coding region	MOWSE score	name	description	GenDB ID
cg0040	123<br>72<br>80<br>88<br>89<br>90		PUTATIVE SECRETED PROTEIN	2631
cg0482	74	gpmA	PHOSPHOGLYCEROMUTASE 1 (EC 5.4.2.1)	127
cg0700	73	guaB3	IMP dehydrogenase / GMP reductase C terminus	3013
cg0755	154	metY	O-acetylhomoserine sulfhydrylase	1136
cg1111	76<br>77<br>79<br>80	eno	ENOLASE (EC 4.2.1.11) (2-PHOSPHOGLYCERATE DEHYDRAT	973
cg1145	85	fum	FUMARATE HYDRATASE (EC 4.2.1.2)	5
cg1380	72	ssuA	ABC-type aliphatic sulfonate transporter, substrate-binding protein	1087
cg1514	70		secreted protein	1513
cg1735	121<br>130<br>141<br>166<br>170<br>173		secreted cell wall-associated hydrolase (invasion-associated protein)	2421
cg1763	101<br>75	sufD	components of an uncharacterized iron-regulated ABC-type transporter	852
cg1774	69	tkt	TRANSKETOLASE (EC 2.2.1.1)	2589
cg1790	67<br>92	pgk	PHOSPHOGLYCERATE KINASE (EC 2.7.2.3)	489
cg1791	104<br>111	gap	GLYCERALDEHYDE-3-PHOSPHATE DEHYDROGENASE (EC 1.2.1)	1677

coding region	MOWSE score	name	description	GenDB ID
cg2052	66<br>74<br>75<br>77		putative secreted protein	524
cg2057	68		putative secreted protein	376
cg2221	111	tsf	TRANSLATION ELONGATION FACTOR TS (EF-TS)	2983
cg2361	75<br>75		Cell division initiation protein - Antigen 84 homolog	2846
cg2401	88		SECRETED PROTEIN NLP/P60 FAMILY, PUTATIVE PEPTIDOGLYCAN LYTIC PROTEIN	4414
cg2591	77	dkgA	2,5-DIKETO-D-GLUCONIC ACID REDUCTASE (EC 1.1.1.-)	2755
cg2833	122<br>136<br>154<br>75<br>81<br>94<br>98	cysK	O-Acetylserine (Thiol)-Lyase	532
cg2840	80<br>82	actA	BUTYRYL-COA:ACETATE COENZYME A TRANSFERASE	2187
cg3049	98	fpr1	NADPH-dependent ferredoxin reductase	277
cg3096	114<br>83		ALDEHYDE DEHYDROGENASE (EC 1.2.1.3)	4002
cg3100	121<br>127<br>141<br>72<br>76	dnaK	Heat shock protein hsp70	2581
cg3115	99	cysD	sulfate adenylyltransferase subunit 2	682
cg3119	72	fpr2	NADPH-dependent ferredoxin reductase	2569
cg3182	69<br>83	cop1	Trehalose corynomycolyl transferase2	3095
