# Supplementary material for: Peak intensity prediction in MALDI-TOF mass spectrometry: A machine learning study to support quantitative proteomics
Source: BMC Bioinformatics. 2008 Oct 20;9:443. doi: 10.1186/1471-2105-9-443 (PMC2600826; doi:10.1186/1471-2105-9-443)
Supplement: Additional file 4 — Lists of the identified proteins. Identified proteins, their coding region, description, MASCOT score, and GenDB [45] ID. Additional information about the MASCOT search parameters that led to the highest score are included: coverage, number of peptides (found and overall), missed cleavages, mass tolerance, modifications, and the number of search parameter sets that led to the identification of the corresponding protein. [file 1471-2105-9-443-S4.pdf]

coding region	MOWSE score	name	description	GenDB ID	coverage	MC	min. mass tolerance	modifications	parameter sets
cg0004	200	dnaN	DNA polymerase III, beta subunit	437	52%: 15/27	1	50ppm	Oxidation (M)	42/42
cg0040	67		putative secreted protein	2631	18%: 6/40	1	50ppm	None	30/42
cg0131	86		putative oxidoreductase	2004	25%: 8/24	1	200ppm	None	23/42
cg0203	77	iolE	putative inosose dehydratase	2355	28%: 8/63	0	200ppm	None	11/42
cg0204	101	iolG	myo-inositol 2-dehydrogenase	1230	35%: 11/68	0	100ppm	Oxidation (M)	35/42
cg0267	114	pat	phenylalanine aminotransferase	3444	30%: 9/39	0	50ppm	Oxidation (M)	38/42
cg0307	88	asd	aspartate-semialdehyde dehydrogenase (EC 1.2.1.11)	254	27%: 6/28	0	100ppm	None	36/42
cg0310	110<br>155<br>332	katA	catalase (EC 1.11.1.6)	1894	31%: 13/29<br>28%: 10/19<br>71%: 34/65	0<br>0<br>1	500ppm<br>100ppm<br>50ppm	Oxidation (M)<br>Oxidation (M)<br>Oxidation (M)	12/42<br>32/42<br>42/42
cg0387	152	adhE	putative zinc-type alcohol dehydrogenase transmemb	2260	47%: 13/49	0	150ppm	Oxidation (M)	39/42
cg0391	99	rmlB2	putative dtdp-glucose 4,6-dehydratase	2555	35%: 9/30	1	150ppm	None	36/42
cg0400	130	adhC	alcohol dehydrogenase, class c (EC 1.1.1.2)	819	47%: 11/58	0	100ppm	Oxidation (M)	36/42
cg0413	83<br>99	cmt1	Trehalose corynomycolyl transferase	243	26%: 9/45<br>29%: 10/45	1<br>1	50ppm<br>50ppm	Oxidation (M)<br>Oxidation (M)	38/42<br>35/42
cg0441	237<br>244	lpd	dihydrolipoamide dehydrogenase (EC 1.8.1.4)	519	37%: 15/21<br>47%: 20/32	0<br>1	100ppm<br>50ppm	Oxidation (M)<br>Oxidation (M)	42/42<br>42/42
cg0482	129	gpmA	phosphoglyceromutase 1 (EC 5.4.2.1)	127	43%: 8/29	0	100ppm	None	42/42
cg0488	89	ppx1	exopolyphosphatase	4068	21%: 6/20	0	100ppm	None	37/42
cg0548	66	menB	dihydroxynaphthonic acid synthase (EC 4.1.3.36)	2502	18%: 6/21	1	100ppm	None	25/42
cg0583	134<br>168<br>259	fusA	elongation factor g	1897	20%: 12/20<br>28%: 16/27<br>40%: 22/50	1<br>1<br>0	50ppm<br>100ppm<br>100ppm	Oxidation (M)<br>Oxidation (M)<br>Oxidation (M)	39/42<br>36/42<br>42/42
cg0587	117<br>120	tuf	elongation factor tu	2252	37%: 9/30<br>37%: 9/28	0<br>0	100ppm<br>100ppm	Oxidation (M)<br>Oxidation (M)	41/42<br>35/42
cg0650	71<br>82		secreted protein	1117	30%: 6/46<br>38%: 6/41	0<br>0	50ppm<br>50ppm	Oxidation (M)<br>Oxidation (M)	35/42<br>31/42
cg0655	110<br>127	rpoA	DNA-directed RNA polymerase alpha subunit	3122	27%: 8/15<br>28%: 9/16	1<br>1	50ppm<br>50ppm	Oxidation (M)<br>Oxidation (M)	42/42<br>42/42
cg0699	202<br>318	guaB2	inositol-monophosphate dehydrogenase (EC 1.1.1.205)	1879	61%: 25/39<br>61%: 32/43	1<br>1	750ppm<br>50ppm	None<br>Oxidation (M)	6/42<br>42/42

coding region	MOWSE score	name	description	GenDB ID	coverage	MC	min. mass tolerance	modifications	parameter sets
cg0700	117	guaB3	IMP dehydrogenase/GMP reductase C terminus	3013	30%: 9/22	1	50ppm	None	42/42
cg0726	85		secreted lipoprotein	444	33%: 5/22	0	50ppm	None	32/42
cg0737	110	metQ	ABC-type methionine transporter, substrate-binding protein	91	45%: 8/52	1	50ppm	None	42/42
	85				42%: 7/70	1	50ppm	None	34/42
cg0750	101	folD	methylenetetrahydrofolate dehydrogenase	2950	32%: 13/28	1	750ppm	None	6/42
cg0755	88	metY	O-acetylhomoserine sulfhydrylase	1136	22%: 8/21	1	150ppm	None	42/42
cg0760	125	prpB2	probable methylisocitric acid lyase	1886	38%: 12/29	1	150ppm	Oxidation (M)	36/42
cg0779	134	trpS	tryptophan tRNA synthetase	1752	32%: 9/31	0	50ppm	None	42/42
cg0792	68		Thioredoxin domain-containing protein	952	31%: 8/46	1	200ppm	None	22/42
cg0802	117	accBC	(Q54119) biotin carboxylase and biotin carboxyl ca		24%: 11/14	1	500ppm	None	12/42
	126			24	35%: 22/35	1	500ppm	Oxidation (M)	12/42
	151			24	41%: 24/38	1	500ppm	Oxidation (M)	12/42
	279			24	38%: 22/28	1	100ppm	Oxidation (M)	42/42
	70			24	15%: 7/24	1	50ppm	None	13/42
cg0835	70	msiK2	ABC-type sugar transport systems, ATPase component	477	18%: 6/28	0	100ppm	Oxidation (M)	7/42
cg0854	66	pmmA	phosphomannomutase (EC 5.4.2.8)	3556	16%: 6/28	0	200ppm	None	9/42
cg0867	98		Ribosome-associated protein Y (PSrp-1)	1647	46%: 8/31	1	150ppm	None	27/42
cg0873	165	aroA	3-phosphoshikimate 1-carboxyvinyltransferase	2244	47%: 15/38	1	100ppm	Oxidation (M)	42/42
	179				43%: 14/30	1	100ppm	Oxidation (M)	42/42
cg0898	104		Pyridoxine biosynthesis enzyme	296	41%: 8/48	0	150ppm	None	24/42
	91				29%: 7/15	1	100ppm	Oxidation (M)	15/42
cg0924	77		ABC-type cobalamin/Fe3+-siderophores transport sys	2856	24%: 5/27	0	50ppm	None	30/42
	88				26%: 5/14	0	50ppm	Oxidation (M)	28/42
	95				32%: 7/44	1	50ppm	None	36/42
cg0967	115	cysQ	3'-Phosphoadenosine 5'-phosphosulfate (PAPS) 3'-phosphatase	1502	36%: 7/26	0	50ppm	None	42/42
cg0984	161	purH	(Q9RHX6) 5'-phosphoribosyl-5-aminoimidazole-4-carb	3001	27%: 11/14	1	50ppm	Oxidation (M)	42/42
	246				39%: 18/24	1	50ppm	Oxidation (M)	42/42
cg1037	69	rpf2	RPF2 precursor, secreted protein	190	20%: 5/14	1	50ppm	None	42/42
cg1111	109	eno	enolase (EC 4.2.1.11) (2-phosphoglycerate dehydrat	973	32%: 11/26	1	500ppm	None	12/42
	127				23%: 7/12	0	100ppm	None	39/42
	131				23%: 7/12	0	50ppm	None	42/42
	142				33%: 10/18	1	100ppm	None	42/42

coding region	MOWSE score	name	description	GenDB ID	coverage	MC	min. mass tolerance	modifications	paramete sets
cg1113	76		conserved hypothetical protein	2358	38%: 6/19	0	500ppm	Oxidation (M)	26/42
cg1133	98	glyA	Serine Hydroxymethyltransferase	2386	41%: 12/48	0	750ppm	None	6/42
cg1145	124	fum	fumarate hydratase (EC 4.2.1.2)	5	36%: 18/43	0	750ppm	Oxidation (M)	6/42
cg1171	130<br>95		Predicted GTPase	3579	37%: 9/36<br>27%: 7/20	0<br>0	50ppm<br>200ppm	Oxidation (M)<br>Oxidation (M)	41/42<br>31/42
cg1206	77	PEP	phosphonomutase or related enzyme	2961	34%: 8/32	1	50ppm	Oxidation (M)	30/42
cg1283	67	aroE2	putative shikimate/quinate 5-dehydrogenase	2344	20%: 4/16	0	50ppm	None	12/42
cg1322	86		conserved hypothetical protein	2682	28%: 5/13	1	50ppm	None	35/42
cg1334	75	lysA	diaminopimelate decarboxylase	582	37%: 11/53	0	750ppm	Oxidation (M)	6/42
cg1343	106	narH	probable respiratory nitrate reductase oxidoreduct	1098	15%: 6/10	0	50ppm	None	42/42
cg1355	127<br>69	prfA	peptide chain release factor 1 (RF-1)	3754	50%: 13/40<br>17%: 4/11	1<br>0	150ppm<br>100ppm	Oxidation (M)<br>None	39/42<br>12/42
cg1365	86	atpH	H+-ATPase delta subunit	693	36%: 8/33	1	150ppm	None	40/42
cg1366	135<br>188<br>196	atpA	probable ATP synthase alpha chain protein	177	26%: 13/32<br>46%: 19/26<br>49%: 23/30	1<br>1<br>1	100ppm<br>750ppm<br>750ppm	None<br>None<br>Oxidation (M)	42/42<br>6/42<br>19/42
cg1367	90<br>96	atpG	ATP synthase gamma subunit (EC 3.6.1.34)	728	23%: 6/21<br>23%: 7/16	0<br>0	100ppm<br>50ppm	None<br>Oxidation (M)	28/42<br>34/42
cg1368	111	atpD	ATP synthase beta subunit	1880	26%: 10/38	0	50ppm	Oxidation (M)	36/42
cg1404	98<br>99	gatA	probable glu-tRNA (GLN) amidotransferase (subunit A)	1443	20%: 6/16<br>31%: 13/30	0<br>0	100ppm<br>750ppm	None<br>Oxidation (M)	42/42<br>6/42
cg1409	80	pfkA	6-phosphofructokinase	392	26%: 8/25	0	500ppm	Oxidation (M)	25/42
cg1418	113		ABC-type cobalamin/Fe3+-siderophores transport system secreted component	2868	31%: 8/37	0	50ppm	None	41/42
cg1420	168<br>177	gatB	probable GLU-tRNA (GLN) amidotransferase subunit B pr	3833	31%: 10/23<br>40%: 13/24	0<br>1	50ppm<br>50ppm	None<br>None	42/42<br>42/42
cg1453	141	leuB	3-isopropylmaltate dehydrogenase (EC 1.1.1.85)	3348	47%: 11/58	0	150ppm	None	35/42
cg1514	91		secreted protein	1513	34%: 9/65	1	150ppm	Oxidation (M)	42/42
cg1531	100<br>174	rpsA	30S ribosomal protein S1	2206	27%: 11/34<br>32%: 17/35	0<br>1	500ppm<br>200ppm	Oxidation (M)<br>Oxidation (M)	11/42<br>42/42
cg1574	80	pheS	phenylalanyl-tRNA synthetase alpha chain	698	19%: 5/12	0	100ppm	Oxidation (M)	13/42
cg1577	80<br>94		putative secreted hydrolase	394	32%: 7/80<br>32%: 7/57	0<br>0	100ppm<br>50ppm	None<br>None	29/42<br>32/42

coding region	MOWSE score	name	description	GenDB ID	coverage	MC	min. mass tolerance	modifications	parameter sets
cg1598	68		sugar phosphatase of the HAD superfamily	2641	15%: 4/11	0	50ppm	None	16/42
cg1630	79		putative signal transduction protein, FHA domain	2573	39%: 5/20	1	50ppm	None	28/42
cg1692	85	pimT	SAM-dependent methyltransferase involved in tRNA-Met maturation	2529	26%: 8/31	1	150ppm	None	30/42
cg1735	145<br>183<br>274		secreted cell wall-associated hydrolase (invasion-associated protein)	2421	26%: 14/43<br>31%: 16/42<br>38%: 24/46	1<br>1<br>1	50ppm<br>50ppm<br>50ppm	Oxidation (M)<br>Oxidation (M)<br>Oxidation (M)	40/42<br>42/42<br>42/42
cg1762	125	sufC	Iron-regulated ABC transporter ATPase subunit	1407	42%: 7/18	0	50ppm	Oxidation (M)	42/42
cg1774	65	tkt	transketolase (EC 2.2.1.1)	2589	16%: 8/32	0	500ppm	Oxidation (M)	6/42
cg1789	113<br>114	tpi	triosephophate isomerase	3400	38%: 9/30<br>38%: 9/32	1<br>1	100ppm<br>50ppm	None<br>None	42/42<br>42/42
cg1790	243	pgk	phosphoglycerate kinase (EC 2.7.2.3)	489	57%: 13/28	0	100ppm	None	42/42
cg1791	102<br>176	gap	glyceraldehyde-3-phosphate dehydrogenase	1677	16%: 8/18<br>53%: 23/45	1<br>1	100ppm<br>750ppm	None<br>Oxidation (M)	29/42<br>6/42
cg1814	101<br>130	carA	carbamoyl phosphate synthase small subunit	3239	34%: 9/31<br>35%: 8/26	0<br>0	100ppm<br>100ppm	Oxidation (M)<br>None	34/42<br>40/42
cg1815	119<br>188<br>68	pyrC	putative dihydroorotase (EC 3.5.2.3)	968	22%: 7/7<br>43%: 16/32<br>29%: 11/33	1<br>0<br>0	100ppm<br>150ppm<br>750ppm	Oxidation (M)<br>Oxidation (M)<br>Oxidation (M)	42/42<br>36/42<br>6/42
cg1817	75	pyrR	Pyrimidine operon attenuation protein/uracil phosphoribosyltransferase	2595	38%: 6/22	1	50ppm	Oxidation (M)	11/42
cg1825	69	efp	Translation elongation factor P/translation initiation factor eIF-5A	4023	28%: 5/14	0	100ppm	Oxidation (M)	13/42
cg1826	313	pepQ	XAA-PRO aminopeptidase (EC 3.4.11.9)	1708	69%: 16/28	0	50ppm	None	42/42
cg1855	124	hisS	histidyl-tRNA synthetase (EC 6.1.1.21)	3461	37%: 14/29	0	750ppm	Oxidation (M)	6/42
cg2057	108		putative secreted protein	376	35%: 9/30	1	50ppm	None	42/42
cg2091	152	ppgK	polyphosphate glucokinase (EC 2.7.1.2)	1389	44%: 8/15	0	50ppm	Oxidation (M)	42/42
cg2103	89	dtxR	iron dependent regulatory protein - DTXR homolog	1654	52%: 12/43	0	750ppm	Oxidation (M)	5/42
cg2117	193	ptsI	phosphoenolpyruvate: sugar phosphotransferase system enzymei	1018	38%: 19/47	1	200ppm	None	30/42
cg2137	65	gluB	glutamate secreted binding protein	2765	35%: 7/62	1	100ppm	Oxidation (M)	9/42
cg2151	142		Similar to phage shock protein A	4362	47%: 12/24	0	150ppm	Oxidation (M)	33/42
cg2217	70	frr	ribosome recycling factor	1690	29%: 5/7	1	100ppm	Oxidation (M)	20/42
cg2221	149<br>85	tsf	translagtion elongation factor TS (EF-TS)	2983	68%: 15/46<br>44%: 8/37	1<br>1	200ppm<br>150ppm	Oxidation (M)<br>None	36/42<br>39/42

coding region	MOWSE score	name	description	GenDB ID	coverage	MC	min. mass tolerance	modifications	parameter sets
cg2222	94	rpsB	30S ribosomal protein S2	3216	32%: 9/26	1	100ppm	Oxidation (M)	32/42
cg2275	156		putative F0F1-type ATP synthase b subunit	3051	54%: 17/69	1	50ppm	None	42/42
cg2291	142	pyk	pyruvate kinase	1271	25%: 10/14	1	150ppm	None	30/42
cg2297	129	hisF	probable cyclase (imidazole glycerol phosphate synthase-subunit)	2718	46%: 9/23	1	100ppm	None	36/42
cg2305	100	hisD	histidinol dehydrogenase (EC 1.1.1.23)	2680	21%: 8/19	1	100ppm	None	26/42
cg2363	68		conserved hypothetical protein	554	44%: 4/25	0	50ppm	Oxidation (M)	9/42
cg2366	121	ftsZ	Cell division GTPase	2356	26%: 8/11	1	50ppm	Oxidation (M)	40/42
cg2391	126	aroG	phospho-2-dehydro-3-deoxyheptonate aldolase	3899	29%: 10/33	0	100ppm	Oxidation (M)	40/42
cg2418	80<br>96	ilvE	branched-chain amino acid aminotransferase	1975	17%: 5/11<br>27%: 7/28	0<br>0	100ppm<br>50ppm	Oxidation (M)<br>Oxidation (M)	21/42<br>19/42
cg2429	117<br>120	glnA	glutamine synthetase I (EC 6.3.1.2)	386	28%: 10/19<br>25%: 9/17	1<br>1	100ppm<br>50ppm	Oxidation (M)<br>None	42/42<br>42/42
cg2466	222	aceE	pyruvate dehydrogenase E1 component (EC 1.2.4.1)	3330	28%: 23/29	1	100ppm	Oxidation (M)	42/42
cg2610	102		ABC-type dipeptide/oligopeptide/nickel transport system, secreted component	2828	22%: 9/45	1	50ppm	None	37/42
cg2658	79	rpi	possible phosphopentose isomerase	3661	45%: 5/29	0	150ppm	None	28/42
cg2692	104		Predicted thioesterase	3890	53%: 8/35	1	100ppm	None	39/42
cg2705	128<br>144<br>151<br>154<br>193<br>194<br>247<br>265<br>75<br>78	amyE	maltose-binding protein precursor	1319	30%: 10/29<br>36%: 14/32<br>46%: 14/66<br>49%: 18/74<br>60%: 21/67<br>58%: 20/58<br>57%: 19/55<br>65%: 21/59<br>21%: 6/10<br>28%: 11/64	0<br>1<br>0<br>1<br>1<br>1<br>1<br>1<br>1<br>1	50ppm<br>50ppm<br>50ppm<br>50ppm<br>100ppm<br>100ppm<br>50ppm<br>100ppm<br>250ppm<br>50ppm	Oxidation (M)<br>Oxidation (M)<br>Oxidation (M)<br>Oxidation (M)<br>Oxidation (M)<br>Oxidation (M)<br>None<br>None<br>None<br>Oxidation (M)	42/42<br>42/42<br>40/42<br>40/42<br>42/42<br>42/42<br>42/42<br>42/42<br>19/42<br>27/42
cg2720	116	lppS	secreted lipoprotein ErfK/YbiS/YcfS/YnhG family	3516	32%: 10/42	1	100ppm	None	39/42
cg2736	74	bcp	probable bacterioferritin comigratory oxidoreductase	187	46%: 5/16	1	50ppm	Oxidation (M)	20/42
cg2751	80		Xanthosine triphosphate pyrophosphatase	3153	40%: 6/28	1	50ppm	None	30/42
cg2830	69	pduO	adenosylcobalamin-dependent diol dehydratase gamma	1034	21%: 4/7	1	100ppm	None	32/42
cg2833	98	cysK	O-Acetylserine (Thiol)-Lyase	532	21%: 6/9	1	100ppm	None	42/42
cg2836	150	sucD	succinyl-CoA synthetase alpha subunit	129	50%: 9/24	0	100ppm	Oxidation (M)	42/42
cg2840	75<br>93<br>97	actA	butyryl-CoA: acetate coenzyme A transferase	2187	27%: 7/19<br>28%: 11/27<br>33%: 10/37	0<br>1<br>0	750ppm<br>150ppm<br>250ppm	None<br>Oxidation (M)<br>Oxidation (M)	9/42<br>36/42<br>24/42

coding region	MOWSE score	name	description	GenDB ID	coverage	MC	min. mass tolerance	modifications	parameter sets
cg2856	148	purM	phosphoribosyl aminoimidazole synthetase	2315	48%: 13/59	0	150ppm	Oxidation (M)	42/42
cg2863	128	purQ	phosphoribosylformyl glycinamidine synthase	82	55%: 8/29	0	100ppm	Oxidation (M)	39/42
cg2865	70	purS	phosphoribosylformyl glycinamidine synthase component	2802	49%: 4/16	1	50ppm	None	23/42
cg2874	126	purC	phosphoribosylaminoimidazole-succinocarboxamide synthase	2484	40%: 10/34	0	100ppm	Oxidation (M)	38/42
cg2891	103	poxB	pyruvate dehydrogenase	3835	16%: 8/15	1	50ppm	None	42/42
cg2900	149<br>67	ddh	mesa-diaminopimelate dehydrogenase	3514	40%: 11/21<br>39%: 10/26	1<br>1	100ppm<br>750ppm	Oxidation (M)<br>Oxidation (M)	42/42<br>6/42
cg3049	130	fpr1	NADPH-dependent ferredoxin reductase	277	45%: 20/30	1	750ppm	Oxidation (M)	12/42
cg3050	90		acyltransferase	4123	35%: 6/24	0	100ppm	Oxidation (M)	29/42
cg3068	123<br>211	fda	fructose-bisphosphate aldolase	875	46%: 9/48<br>66%: 13/53	0<br>0	150ppm<br>50ppm	None<br>None	28/42<br>42/42
cg3079	129	clpB	probable ATP-dependent protease (heat shock protein)	936	23%: 19/40	1	150ppm	Oxidation (M)	33/42
cg3096	188		aldehyde dehydrogenase (EC 1.2.1.3)	4002	47%: 20/54	1	150ppm	Oxidation (M)	36/42
cg3100	245<br>257<br>69	dnaK	Heat shock protein hsp70	2581	39%: 15/29<br>41%: 16/28<br>23%: 8/34	0<br>0<br>0	100ppm<br>150ppm<br>500ppm	None<br>None<br>None	42/42<br>36/42<br>5/42
cg3182	159	cop1	Trehalose corynomycolyl transferase	3095	25%: 13/61	0	50ppm	None	42/42
cg3197	102	psp5	Putative secreted protein	2257	15%: 8/44	0	50ppm	None	38/42
cg3214	66		conserved hypothetical protein	762	30%: 7/52	1	50ppm	None	21/42
cg3264	106		conserved hypothetical protein	2594	38%: 15/37	1	100ppm	Oxidation (M)	37/42
cg3319	70		Uncharacterized enzyme related to sulfurtransferases	1434	18%: 8/33	1	250ppm	None	24/42
cg3350	122	hpaG	putative bifunctional enzyme 2-hydroxyhepta-2,4-diene-1, 7-dioatesomerase5-carboxymethyl-2-oxo-hex-3	3279	40%: 8/37	0	50ppm	None	41/42
cg3423	102	trxC	thioredoxin	3758	54%: 6/19	1	100ppm	None	41/42
