# Supplementary material for: Peak intensity prediction in MALDI-TOF mass spectrometry: A machine learning study to support quantitative proteomics
Source: BMC Bioinformatics. 2008 Oct 20;9:443. doi: 10.1186/1471-2105-9-443 (PMC2600826; doi:10.1186/1471-2105-9-443)
Supplement: Additional file 5 — Raw spectra preprocessing and peak extraction details. [file 1471-2105-9-443-S5.pdf]

Preprocessing details

The following steps are performed by the peak extraction software:

De-noising and baseline correction Noise filtering is done with a Savitzky-Golay filter of length 17 and degree 4 [SG64]. These parameters have been selected by manual inspection in a way that allows the subsequent maxima and minima selection to work properly and not lower the peak tips too much. A baseline correction is applied by calculating a list of maxima and minima on the filtered spectra, controlled by parameters for the minimum and maximum width of a peak. The list of minima is used to estimate the baseline, which is substracted from the de-noised spectrum.

Eliminate noise peaks The list of maxima is filtered for noise peaks by the following algorithm, where we set w to 500 Da and $\xi = 3$:

1. set a window size w
2. for each window do
   a) sort the maxima by their height
   b) calculate b as the mean of the ordered list after trimming away the upper 50% and the lower 25% of the values
   c) set the threshold $\theta = b\ \xi$, where ξ is a user defined parameter
   d) discard all peaks with height below θ.

The multiplier ξ was chosen after testing different values by visual inspection of the spectra. Peaks still present after this cleaning step are considered for the consecutive steps (isotopic deconvolution and peak matching).

Isotopic deconvolution Isotopic deconvolution was carried out as follows: We calculate a the theoretical isotope pattern of an average protein in 500 Da steps throughout the appropriate mass range. For peaks that lie between these calculated points, the isotope pattern is linearly interpolated. The nth isotope peak for a monoisotopic peak at mass m with intensity i is denoted $h(i, m, n)$. We iterate through the list of maxima in ascending order of mass-per-charge ratio.

For each peak

1. denote its mass m_1 and its intensity i_1, and assume that it is a monoisotopic peak.
2. set $n = 2$
3. **while** (there is a peak inside $[m_{n-1} + 0.9, m_{n-1} + 1.1]$ Da)
   - denote this peak p_n with intensity i_n, assume it to be the nth isotopic peak

- if($i_n < 0.01\ i_1$): continue to next peak, beginning with step 1
- calculate the theoretical intensity of the second isotopic peak $\hat{i}_n = h(i_1, m_1, n)$.
- update i_2 and i_1:
$$i_n^{new} = i_n - \hat{i}_n, \quad i_1^{new} = i_1 + \hat{i}_n$$
- $n = n + 1$

Theoretical digestion Now having a list of deconvoluted monoisotopic peaks with summed intensities, the protein sequence from the MASCOT peptide mass fingerprint identification is used to perform a theoretical tryptic digestion on it. As a result, a list of peptides is retrieved. Cleavages are set at positions after a "K" or "R" if not a "P" is following. Only peptides that would result from a perfect digestion are calculated. We calculate monoisotopic masses of these theoretical peaks.

Peak matching Using the masses from the theoretical digestion we look for matches all over the spectrum. The matched peak's intensity is then assigned to the peptide sequence and the spectrum currently under consideration. We allow for mass errors of up to 1.0 Da to consider a peak a match. Spot checks in the resulting mass error in the matched peak lists showed that this large an error actually occurs for large masses, but except for very few matches, the errors increase towards larger masses, suggesting that the matches are sensible even though the calibration was not good. Mismatched peptides (i.e. mass error non-monotonic) mostly occur in the area below 800 Da where most of the matrix noise peaks are expected. In case of multiple peaks being in the allowed window, the one with the lowest error (i.e. the nearest peak) is chosen as a match. We do not choose the peak with the highest intensity since we do not want to assume any knowledge about the intensities which we do not have. The highest peak does not neccessarily have to be the correct match. For A, a cystein mass of 103.009184 was used, while for B we use 160.030648, which is the mass of carbamidomethylated cystein.

The mass ranges for the peak extraction are $[650, 3118]$ for A and $[800, 3578]$ for B.

References

[SG64] A. Savitzky and J. E. M. Golay. Smoothing and differentiation of data by simplified least squares procedures. *Anal. Chem.*, 36:1627 – 1639, 1964.
