# Supplementary material for: Peak intensity prediction in MALDI-TOF mass spectrometry: A machine learning study to support quantitative proteomics
Source: BMC Bioinformatics. 2008 Oct 20;9:443. doi: 10.1186/1471-2105-9-443 (PMC2600826; doi:10.1186/1471-2105-9-443)
Supplement: Additional file 9 — Details of the forward stepwise selection process. Features selected as well as performance values for each run of the forward stepwise selection process are shown in separate tables for selection in aa and seq. [file 1471-2105-9-443-S9.pdf]

r / rss	step 1	step 2	step 3	step 4	step 5	step 6	step 7	step 8	step 9	step 10	step 11	step 12
0.49 / 502	GARJ730101	TANS770108	VASM830103	GB500								
0.55 / 457	ARGP820102	CHOC760102	FAUJ880111	TANS770105	NADH010106	WILM950102	TANS770105	R	GB500			
0.49 / 496	FAUJ880111	ROBB760109	R	GB500								
0.51 / 489	ISOY800102	QIAN880112	VASM830103	NADH010106	GB500							
0.54 / 466	ISOY800103	VASM830103	NADH010106	QIAN880112	WILM950102	R	GB500					
0.56 / 454	FAUJ830101	LEVM780106	OOBM770105	PALJ810114	QIAN880127	RACS820106	RACS820107	VASM830103	NADH010106	GB500		
0.54 / 470	BUNA790103	CHAM830101	CHOP7802010	WOLS870101	OOBM770104	VASM830103	R	GB500				
0.52 / 484	FAUJ880111	QIAN880123	ROBB760109	WOLS870101	R	GB500						
0.48 / 505	PRAM900101	RICJ880114	SNEP660103	GB500								
0.49 / 502	VASM830103	GB500										
0.54 / 466	QIAN880103	QIAN880115	QIAN880123	QIAN880132	QIAN880103	QIAN880104	R	GB500				
0.50 / 495	QIAN880133	VASM830103	NADH010106	GB500								
0.52 / 481	RICJ880115	ROBB760102	VENT840101	NADH010106	R	GB500						
0.56 / 454	CHOP780214	GEIM800105	OOBM770104	VASM830103	NADH010106	WILM950102	GUOD860101	BASU050101	R	GB500		
0.56 / 454	GEIM800109	NOZY710101	RACS820114	RICJ880114	ROSM880103	SIMZ760101	TANS770108	VASM830103	AURR980102	KIMC930101	WILM950102	GB500
0.54 / 471	VASM830103	NADH010105	WILM950102	GEOR030103	NADH010106	GB500	R					
0.53 / 474	FAUJ880111	ISOY800103	ZIMJ680101	NADH010101	NADH010106	WILM95010102	R	GB500				
0.55 / 457	FAUJ880111	NAKH920108	RICJ880104	RICJ880105	ROBB760109	AURR980111	WILM950103	R	GB500			
0.51 / 484	CHAM830103	FAUJ880111	VASM830103	R	GB500							
0.47 / 509	PUNT030101	R	GB500									

Table 0.1: Feature selection details: Features from the *aa* feature set selected in each run (rows) in the order of their stepwise addition to the selection. The step numbers show the order of selection. The Pearson's correlation is denoted by r^2, and rss is the residual sum of squares. The description of features can be found with the Amino Acid Index Database by Kawashima *et al.* (*Nucleic Acids Res 1999*: **27**:368–369).

r / rss	step 1	step 2	step 3	step 4	step 5	step 6	step 7	step 8	step 9	step 10	step 11
4 runs [0.28 / 481]	R	M	F	FFT							
4 runs [0.28 / 475]	R	M	F	GFG							
2 runs [n/a]	R	M	F	YTe							
1 runs [n/a]	R	M	F	DYN							
2 runs [n/a]	R	M	F	DLH							
2 runs [n/a]	R	M	F	P	DLH	TSD					
3 runs [0.32 / 451]	R	F	QQ	GT	HP	ID	MT	AFN	QAK	QSV	LHD
1 runs [n/a]	R	F	MT	EM	SV	KMM	AGL				
1 runs [n/a]	C	K	MF	AGL	QSV	PEC	aSL				

Table 0.2: Feature selection details: Features from the *seq* feature set selected in different runs (rows) in the order of their stepwise addition to the selection. The step numbers show the order of selection. The Pearson's correlation is denoted by r^2, and rss is the residual sum of squares.
